# Upright versus lying down position in second stage of labour in nulliparous women with low dose epidural: BUMPES randomised controlled trial

**DOI:** 10.1136/bmj.j4471

**Published:** 2017-10-18

**Authors:** The Epidural and Position Trial Collaborative Group

## Abstract

**Objective** To determine whether being upright in the second stage of labour in nulliparous women with a low dose epidural increases the chance of spontaneous vaginal birth compared with lying down.

**Design** Multicentre pragmatic individually randomised controlled trial.

**Setting** 41 UK hospital labour wards.

**Participants** 3093 nulliparous women aged 16 or older, at term with a singleton cephalic presentation and in the second stage of labour with epidural analgesia.

**Interventions** Women were allocated to an upright or lying down position, using a secure web based randomisation service, stratified by centre, with no masking of participants or clinicians to the trial interventions.

**Main outcome measures** The primary outcome was spontaneous vaginal birth. Women were analysed in the groups into which they were randomly allocated, regardless of position recorded at any time during the second stage of labour (excluding women with no valid consent, who withdrew, or who did not reach second stage before delivery). Secondary outcomes included mode of birth, perineal trauma, infant Apgar score <4 at five minutes, admission to a neonatal unit, and longer term included maternal physical and psychological health, incontinence, and infant gross developmental delay.

**Results** Between 4 October 2010 and 31 January 2014, 3236 women were randomised and 3093 (95.6%) included in the primary analysis (1556 in the upright group and 1537 in the lying down group). Significantly fewer spontaneous vaginal births occurred in women in the upright group: 35.2% (548/1556) compared with 41.1% (632/1537) in the lying down group (adjusted risk ratio 0.86, 95% confidence interval 0.78 to 0.94). This represents a 5.9% absolute increase in the chance of spontaneous vaginal birth in the lying down group (number needed to treat 17, 95% confidence interval 11 to 40). No evidence of differences was found for most of the secondary maternal, neonatal, or longer term outcomes including instrumental vaginal delivery (adjusted risk ratio 1.08, 99% confidence interval 0.99 to 1.18), obstetric anal sphincter injury (1.27, 0.88 to 1.84), infant Apgar score <4 at five minutes (0.66, 0.06 to 6.88), and maternal faecal incontinence at one year (1.18, 0.61 to 2.28).

**Conclusions** Evidence shows that lying down in the second stage of labour results in more spontaneous vaginal births in nulliparous women with epidural analgesia, with no apparent disadvantages in relation to short or longer term outcomes for mother or baby.

**Trial registration** Current Controlled Trials ISRCTN35706297.

## Introduction

As the most effective form of pain relief in labour, epidural analgesia is chosen by approximately 30% of women in the UK each year, and this proportion has remained relatively stable over the past decade.^1^ ^2^ Epidural analgesia leads to prolongation of the second stage of labour (from full dilation of the cervix until birth) and an increased risk of instrumental vaginal delivery. However, this evidence comes mostly from trials that used epidural techniques which cause dense neuraxial blockade.^3^ Epidurals that use low dose local anaesthetic in combination with opioids result in a lower risk of instrumental vaginal delivery, but the rate of such delivery is still higher than among women with no epidural.^4^ ^5^ Maternal position during the second stage of labour has been suggested to affect the risk of instrumental vaginal delivery. A Cochrane review of position in the second stage of labour in women without epidural showed a reduction in instrumental vaginal delivery in the upright group, although the quality of the included trials was reported to be generally poor.^6^ Maternal mobility is limited with dense neuraxial blockade. Low dose epidurals preserve motor function, allowing greater mobility throughout labour and enabling women to adopt upright positions. A Cochrane review of position in the second stage of labour for women with epidural analgesia was published in 2017, after the current (Birth in the Upright Maternal Position with Epidural in Second stage: BUMPES) trial was started. This review included trials that compared upright with recumbent positions and suggested no effect. The risk ratio of operative birth (caesarean section or instrumental vaginal delivery) reported in the five included trials, comprising 879 women in total, was 0.97 (95% confidence interval 0.76 to 1.25).^7^ In this group of women therefore, the debate remains about whether an upright posture in the second stage of labour increases the incidence of spontaneous vaginal birth.^8^ ^9^

The aim of the BUMPES trial was to evaluate whether, in nulliparous women with low dose epidural analgesia, being upright during the second stage of labour increased the chance of spontaneous vaginal birth, compared with lying down.

## Methods

### Study design and participants

This was a pragmatic randomised controlled trial carried out in UK maternity units. Women were eligible for the trial if they were 16 years or older, were at 37 weeks or more gestation, nulliparous (no previous birth ≥24^+0^ weeks’ gestation), had a singleton cephalic presentation, and intended to have a spontaneous vaginal birth. They had to be in the second stage of labour, have a low dose epidural in situ (administered according to local unit protocol) during the first stage of labour that provided effective pain relief, and be able to understand documents in English and provide written answers in English.

Women were provided with written information about the trial during pregnancy and again in labour. They could give written informed consent during the first stage of labour but were not eligible to be randomised until the second stage of labour had been confirmed. Diagnosis was based on usual clinical criteria, either when the cervix was fully dilated on a vaginal examination (no additional vaginal examinations were specified as part of the trial protocol) or when the presenting part was visible.

### Randomisation and masking

Women were randomised to the allocated intervention (allocation ratio 1:1) using a secure web based central randomisation service hosted by the National Perinatal Epidemiology Unit Clinical Trials Unit, University of Oxford. To ensure that the recruiting staff could not reliably predict the next allocation, the randomisation schedule used random permuted blocks of sizes 2, 4, 6, 8, and 10, randomly selected according to the ratio specified by Pascals’ triangle (1:4:6:8:10). Stratification was by centre. Owing to the nature of the intervention, it was not possible to mask the women or clinicians to the trial intervention.

### Procedures

Women were allocated to a policy of an upright position, which would maintain the pelvis in as vertical a plane as possible during the second stage of labour, with the intention of continuing the allocated position until the birth (this could include walking, standing, sitting out of bed, supported kneeling, bolt upright in an obstetric bed, or any other upright position for as much of the second stage as possible); or a lying down position (left or right lateral, to prevent aorto-caval compression, with up to 30 degrees inclination of the bed), which would maintain the pelvis in as horizontal a plane as possible during the second stage of labour, with the intention of continuing the allocated position until the birth. Because midwives in the UK routinely provide care for women in labour, they administered the intervention. Before the start of the trial, the midwives received training to emphasise the importance of supporting mothers in their allocated position, especially for the passive second stage of labour (the period before pushing commences, which can last one to two hours). As this trial was pragmatic we expected that there would be “unavoidable” reasons for changing maternal position—for example, fetal distress, fetal blood sampling, or to help improve pushing in the active second stage. Women were free to change position if they wished at any stage after trial entry.

From hospital notes we collected information at trial entry about maternal characteristics (including index of multiple deprivation,^10^ an area measure of deprivation derived from the mother’s postcode), pregnancy complications, and progress of labour before trial entry. Data on adherence to the allocated intervention were collected every 15 minutes when the attending midwife recorded what position the woman was in “for the majority of the time since the last assessment” and, if this position had changed from the allocated position, recorded the reasons for this change. Information on clinical outcome after birth as well as neonatal outcomes and hospital inpatient stay was collected after the birth from hospital records. As soon as possible after delivery, the woman was asked to complete a one page questionnaire about satisfaction with her birth experience (see supplementary appendix). Women with infants who survived and resided with them were followed up at one year using a self administered postal questionnaire asking about specific health problems and their general health and wellbeing, as well as that of their baby.

### Outcomes

The primary outcome measure was spontaneous vaginal birth. Secondary short term outcomes were instrumental vaginal delivery (forceps and ventouse), caesarean section, augmentation of labour, major interventions to maintain blood pressure (eg, vasopressors), hypotension (systolic blood pressure <100 mm Hg before delivery), application of fetal scalp electrode, fetal blood sampling, total doses of epidural local anaesthetic and opioids administered after randomisation, duration of active second stage (time from pushing to delivery), duration of second stage of labour (time from randomisation to delivery), additional anaesthesia used for operative delivery, active management of the third stage, episiotomy, pain during delivery (assessed using a visual analogue scale, with 0 as no pain and 10 as worst pain imaginable), genital tract trauma, manual removal of the placenta, primary postpartum haemorrhage requiring blood transfusion, duration of maternal inpatient stay after delivery, and satisfaction with the experience of birth. For the neonate, secondary outcomes were metabolic acidosis (cord artery pH <7.05 in second stage, with base deficit ≥12 mmol/L), presence of meconium stained liquor, Apgar score <4 at five minutes, resuscitation at birth, skin to skin contact within the first hour of birth, initiation of breast feeding within the first hour of birth, duration of neonatal inpatient stay, and admission to neonatal unit and duration of stay. Secondary outcomes at one year for the mother were urinary incontinence, faecal incontinence, other bowel problems, dyspareunia, general physical and psychological health, and health related quality of life measured with the EQ-5D-3L and SF-12 instruments.^11^ ^12^ Secondary outcomes at one year for the infant were major morbidity (eg, gross neurodevelopmental delay, including cerebral palsy) and hospital admissions.

### Statistical analysis

Assuming a rate for the primary outcome of spontaneous vaginal birth of 55% in the control group (derived from data published from the COMET trial^4^) and a two sided 5% significance level, we required a sample size of 3000 women. This had 90% power to detect an absolute difference of 6% in the spontaneous vaginal birth rate between the two policies, equivalent to a risk ratio of 1.11. On collation of the pilot data for an interim analysis presented to the independent data monitoring committee in 2011, it was recognised that the combined primary outcome event rate was lower than anticipated, at 34%, 95% confidence interval 26% to 42% (based on 49/145 events, combining upright and lying down groups). With a reduction in the control group event rate (from an anticipated 55% to between 30% and 40%), keeping the sample size fixed at 3000 meant that a risk ratio of between 1.13 and 1.19 would be detectable, equivalent to an absolute risk increase of 5% to 6%. Although power was insufficient to detect a risk ratio as small as the planned 1.11, the absolute risk detectable was similar and the trial steering committee agreed that changes to the target sample size were unnecessary.

Before analysis of the trial data the trial steering committee developed and approved a detailed statistical analysis plan. Women were analysed in the groups into which they were randomly allocated, regardless of position recorded at any time during the second stage of labour (an intention to treat analysis). We excluded women from the analysis if a valid consent form was not received by the central study team, consent to use their data was withdrawn, they were not in second stage of labour when randomised and did not reach second stage before delivery, or they were not in labour or without an epidural in place at the time of randomisation. All comparative analyses were performed using generalised linear models with centre as a random intercept. Binary outcomes were analysed using log binomial regression models, and results are presented as adjusted risk ratios with corresponding confidence intervals. Where possible we analysed continuous outcomes using linear regression models, and results are presented as adjusted differences in means with associated confidence intervals. Unadjusted Hodges-Lehmann median differences (plus confidence intervals) for skewed continuous variables are presented. The geometric mean indicates the central tendency or typical value of a set of numbers by using the product of their values (as opposed to the arithmetic mean which uses their sum) and is used for summarising skewed data. Comparative analysis uses a ratio of the geometric means instead of the mean difference and therefore a ratio of 1 indicates no difference between the groups. For the primary outcome we present 95% confidence intervals, and for all other outcomes 99% confidence intervals to allow for cautious interpretation of the results.^13^

Positions recorded were categorised according to whether the women were lying down, upright, or in “other” positions for each 15 minute interval. Positions recorded as lithotomy were categorised as “lying down” since the pelvis is in a horizontal position. A summary of adherence to allocated position is reported by trial arm for the passive second stage (ie, before pushing commenced), the active second stage (ie, pushing), and the whole of the second stage. Summaries of adherence data were calculated as the proportion of 15 minute intervals a woman spent in the position to which she was allocated out of the total number of 15 minute intervals recorded in the passive, active, or whole of the second stage of labour. Medians and interquartile ranges are presented owing to the skewed distribution of the data. Hodges-Lehmann differences in medians with corresponding 95% confidence intervals are presented by randomised group. The trial statistician and an independent clinical assessor reviewed and classified all reasons for changes in position as avoidable or unavoidable. Periods where changes to a non-allocated position were considered necessary for unavoidable reasons were treated as adherent.

The self completed maternal satisfaction questionnaire included a question about what position the women were in for most of the time during the passive and active stages of labour, with responses “lying down,” “upright,” “other,” and “can’t remember.” We summarise these data by trial arm using counts and percentages.

To examine whether the effect of the policy of position during the second stage of labour was consistent across prespecified subgroups, we undertook the following subgroup analyses: gestational age (37^+0^ to 38^+6^; 39^+0^ to 40^+6^; and ≥41^+0^); maternal age (≤24, 25-29, 30-34, ≥35); augmentation with oxytocin in the first stage of labour (yes or no); and index of multiple deprivation (population based fifths 1 to 5).

We further adjusted the analysis of the primary outcome to investigate the impact of known prognostic factors (age, ethnicity, diagnosis of delay, onset of labour—induced versus spontaneous).

On a restricted dataset we carried out a sensitivity analysis on the one year maternal outcomes, excluding all women who were pregnant or had had another child at the time of completing the one year follow-up questionnaire.

The time from randomisation to trial entry, and all other durations, are prone to errors because of time differences recorded in different locations of the maternity units. The time of randomisation is accurate as this was recorded by the computer server providing the randomisation service. However, all other times depended on the accuracy of the clocks in the different locations. For example, the clock on the central midwifery station might have read a slightly different time to that in the labour room, and these might both have been different from the clock in theatre. Many relatively minor problems occurred with derived duration variables in the dataset (eg, negative values), suggesting variation in actual time recorded between different settings. Stata version 13 was used for all analyses.

The trial was overseen by a trial steering committee and an independent data monitoring committee. The data monitoring committee used the Haybittle-Peto approach to stopping guidelines for interim analyses using three standard errors as the cut-off for consideration of early cessation, preserving the type 1 error across the trial.^14^

### Patient and public involvement

The public was involved throughout the design, conduct, analysis, and interpretation of this trial. One patient and public involvement co-investigator (MN) attended all the planning meetings and was involved in drafting the funding application, developing the detailed trial protocol and data collection forms, conducting the trial meetings, and writing the report and paper. MN took a lead in helping the team develop participant information leaflets to be used in the antenatal period and at the time of labour, as well as helping plan dissemination activities and drafting and developing the summary information for the public.

## Results

Between 4 October 2010 and 31 January 2014, 3236 women were randomised to the BUMPES trial from 41 participating centres (fig 1 and see supplementary table). This deliberate over-recruitment was to compensate for women being excluded from the analysis. A total of 143/3236 women (4.4%) were excluded from the analysis of the primary outcome (fig 1). Most of these exclusions were because of missing or incomplete consent forms. For 32 women, exclusion was because they were randomised in error. Data at the time of birth were available for 100% of women recruited and analysed. Follow-up data at one year were received from 1892/3093 (61.2%) women. The median time to completion of the questionnaire from birth was 11.9 months (interquartile range 11.7-12.7) in each group.

**Figure f1:**
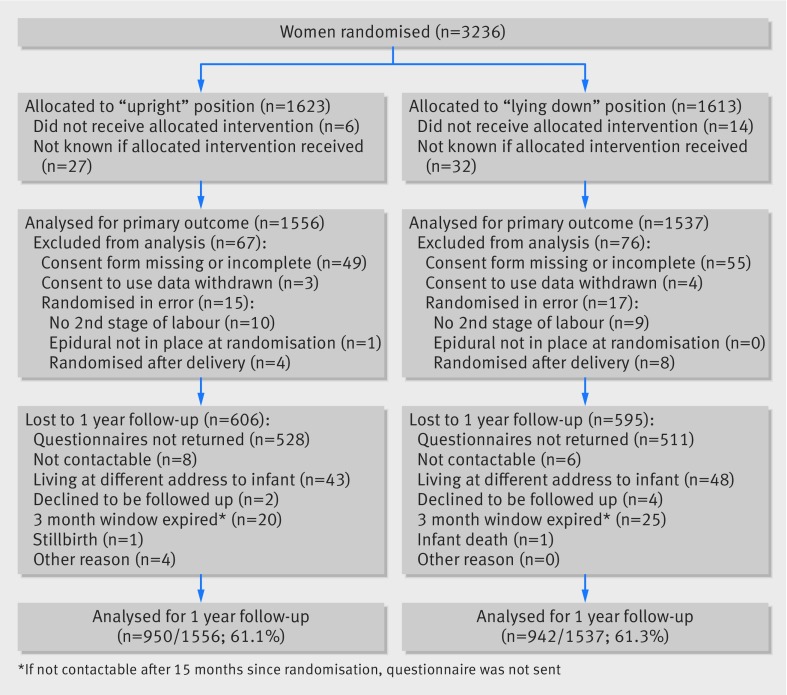
**Fig 1** Flow of participants through trial

Baseline characteristics were broadly similar between the two arms of the trial (table 1). Most of the women in both arms were between 37 and 41 completed weeks of pregnancy, although 7.5% (231/3093) of women were 42 weeks or more. The majority of women participating in the trial were of white ethnic origin and the mean body mass index at booking was just over 25 kg/m^2^. Approximately 40% of women had their labour induced. About 80% of women could perform a straight leg raise at the time of trial entry suggesting that they had reasonable mobility with their epidural analgesia. There was an apparent disparity between the two groups in the position of the women at the time of trial entry; a greater proportion of women who were lying down in the group allocated lying down compared with those in the upright position. The way these data were requested is likely to have led to misclassification of this variable, in that midwives may have recorded the position of the women at the time of allocation (ie, after they had already assumed the allocated position). As all other characteristics of the women were similar at baseline it seems unlikely that this represents the true position at the time of randomisation; rather it is a combination of this plus actual allocation.

**Table 1 tbl1:** Baseline characteristics of analysis population. Values are numbers (percentages) unless stated otherwise

Characteristics	Upright (n=1556)	Lying down (n=1537)
Mean (SD) maternal age (years)	28.4 (5.7)	28.4 (5.6)
Age group (years):		
<20	111 (7.1)	99 (6.4)
20-24	303 (19.5)	292 (19.0)
25-29	437 (28.1)	463 (30.1)
30-34	488 (31.4)	482 (31.4)
35-39	182 (11.7)	161 (10.5)
≥40	34 (2.2)	40 (2.6)
Mean (SD) gestational age at entry (weeks):	40.4 (1.2)	40.4 (1.2)
37^+0^-39^+6^	482 (31.0)	500 (32.6)
40^+0^-41^+6^	955 (61.5)	921 (60.0)
≥42^+0^	116 (7.5)	115 (7.5)
Index of multiple deprivation (fifth):		
First (least deprived)	205 (16.0)	204 (16.0)
Second	182 (14.2)	201 (15.7)
Third	246 (19.2)	235 (18.4)
Fourth	349 (27.2)	345 (27.0)
Fifth (most deprived)	299 (23.3)	294 (23.0)
Wales—not derived	224	217
Postcode missing	51	41
Ethnic group:		
White	1305 (84.5)	1275 (83.5)
Indian	48 (3.1)	57 (3.7)
Pakistani	26 (1.7)	30 (2.0)
Bangladeshi	6 (0.4)	3 (0.2)
Black African	28 (1.8)	30 (2.0)
Black Caribbean	14 (0.9)	11 (0.7)
Any other ethnic group	117 (7.6)	121 (7.9)
Mean (SD) body mass index (at booking visit)	25.5 (5.4)	25.2 (5.3)
Height and/or weight not known	65	60
Woman with female genital mutilation	6 (0.4)	1 (0.1)
Onset of labour:		
Spontaneous	941 (60.6)	904 (58.9)
Induced	613 (39.5)	632 (41.2)
Duration of first stage (mins):		
Median (interquartile range)	510 (360-715)	495 (350-705)
Geometric mean	484.9	481.9
Diagnosis of pre-eclampsia	52 (3.4)	52 (3.4)
Continuous electronic fetal monitoring	1485 (95.5)	1470 (95.8)
Diagnosis of delay requiring intervention	796 (51.2)	770 (50.2)
Systemic opioids given before epidural	442 (28.4)	435 (28.3)
Systemic opioids given:		
Pethidine	353 (79.9)	330 (75.9)
Diamorphine	77 (17.4)	88 (20.2)
Remifentanil	3 (0.7)	4 (0.9)
Morphine	0 (0.0)	0 (0.0)
Meptid	12 (2.7)	17 (3.9)
Epidural technique:		
Epidural	1492 (96.0)	1481 (96.4)
Combined spinal epidural	62 (4.0)	55 (3.6)
Epidural maintained with PCEA/infusion	1224 (80.6)	1196 (79.9)
Woman’s pain score for last contraction*:		
Median (interquartile range)	10 (0-30)	10 (0-38)
Missing	162	184
Able to perform straight leg raise	1162 (78.7)	1152 (80.2)
Missing	79	101
Position before study entry:		
Lying down	432 (29.0)	546 (37.7)
Upright	977 (65.6)	832 (57.4)
Lithotomy	5 (0.3)	6 (0.4)
Semi-recumbent	58 (3.9)	53 (3.7)
Other	17 (1.1)	12 (0.8)
Missing	67	88
Median (interquartile range) time from 2nd stage determined by VE to study entry (mins)	16 (9-30)	16 (8-30)
Apparently randomised before diagnosis of second stage†	70	79
Time apparently >180 mins‡	6	7
Median (interquartile range) time from study entry to start of recording positions (mins)	1 (−2-6)	1 (−3-7)
Time from study entry to recording position >15 mins‡	154 (11.9)	150 (11.6)
Time apparently >15 mins before study entry†	227	218
Mean (SD) baby’s birth weight (g)§	3500 (450)	3488 (442)

PCEA=patient controlled epidural anaesthesia; VE=vaginal examination.

Missing data are <3% unless otherwise presented. There were no apparent differences in missing data between trial groups.

*Measured using a visual analogue scale with 0 as no pain and 10 as worst pain imaginable.

†Values set to missing for calculation of median and interquartile range. The term “apparently” reflects uncertainty about some time durations (see Methods).

‡Values included for calculation of median and interquartile range.

§Measured after study entry but not an outcome.

Adherence to the intervention was generally good. The median proportion of time spent in the allocated position during the passive second stage (before pushing commenced) was 1.0 (interquartile range 1.0-1.0) in the upright group and 1.0 (0.67-1.0) in the lying down group (median difference 0, 95% confidence interval 0 to 0). As anticipated, adherence was poorer in the active second stage, with the median proportion of time spent in the allocated position 0.88 (0.60-1.0) in the upright group and 0.75 (0.38-1.0) in the lying down group (median difference 0, 0 to 0) (table 2, fig 2). The information provided by women about their position in labour was broadly similar to this, with 75.8% (794/1047) of women in the upright group stating that they were mostly in the upright position during the passive stage and 72.3% (752/1040) of women in the lying down group stating they were mostly lying down. In the active stage, 72.5% (745/1028) of women in the upright group recalled being mostly upright, and 63.7% (652/1024) of women in the lying down group recalled mostly lying down (table 2).

**Table 2 tbl2:** Proportion of time spent in allocated position. Values are numbers (percentages) unless stated otherwise

Outcomes	Upright (n=1556)	Lying down (n=1537)	Median difference (95% CI)
No, median (interquartile range) during passive 2nd stage^*^	946, 1.0 (1.0-1.0)	960, 1.0 (0.67-1.0)	0 (0 to 0)
Missing:			
No passive time periods recorded	320	314	
Time from study entry to start of recording positions >15 mins	227	217	
Pushing or birth dates/times not recorded	13	10	
Position times not recorded	50	36	
No, median (interquartile range) during active 2nd stage†	1255, 0.88 (0.60-1.0)	1255, 0.75 (0.38-1.0)	0 (0 to 0)
No active time periods recorded	11	19	
Time from study entry to start of recording positions>15 mins	227	217	
Pushing or birth dates/times not recorded	13	10	
Position times not recorded	50	36	
No, median (interquartile range) during whole 2nd stage^‡^	1274, 0.88 (0.67-1.0)	1284, 0.78 (0.50-1.0)	0 (0 to 0)
Time from study entry to start of recording positions >15 mins	227	217	
Birth dates/times not recorded	1	0	
Position times not recorded	54	36	
Reason for change from allocated position:			
Passive stage:	201	343	
Clinical	94 (50.0)	78 (24.5)	
Non-clinical	77 (41.0)	218 (68.3)	
Clinical and non-clinical	17 (9.0)	23 (7.2)	
Active stage:	699	981	
Clinical	416 (60.6)	298 (31.1)	
Non-clinical	136 (19.8)	368 (38.5)	
Clinical and non-clinical	135 (19.7)	291 (30.4)	
Whole of second stage:	788	1082	
Clinical	435 (56.6)	306 (28.9)	
Non-clinical	164 (21.3)	419 (39.5)	
Clinical and non-clinical	170 (22.1)	335 (31.6)	
Maternal reported adherence:			
Passive stage:			
Mostly lying down	226 (21.6)	752 (72.3)	
Mostly upright	794 (75.8)	242 (23.3)	
Other	24 (2.3)	35 (3.4)	
Can’t remember	3 (0.3)	11 (1.1)	
Missing	161	125	
Form not completed	348	372	
Active stage:			
Mostly lying down	202 (19.7)	652 (63.7)	
Mostly upright	745 (72.5)	281 (27.4)	
Other	78 (7.6)	75 (7.3)	
Can’t remember	3 (0.3)	16 (1.6)	
Missing	180	141	
Form not completed	348	372	

Missing data are <3% unless otherwise presented. There were no apparent differences in missing data between trial groups.

*Time from study entry to when pushing commenced.

†Time from when pushing commenced until birth.

‡Time from study entry until birth.

**Figure f2:**
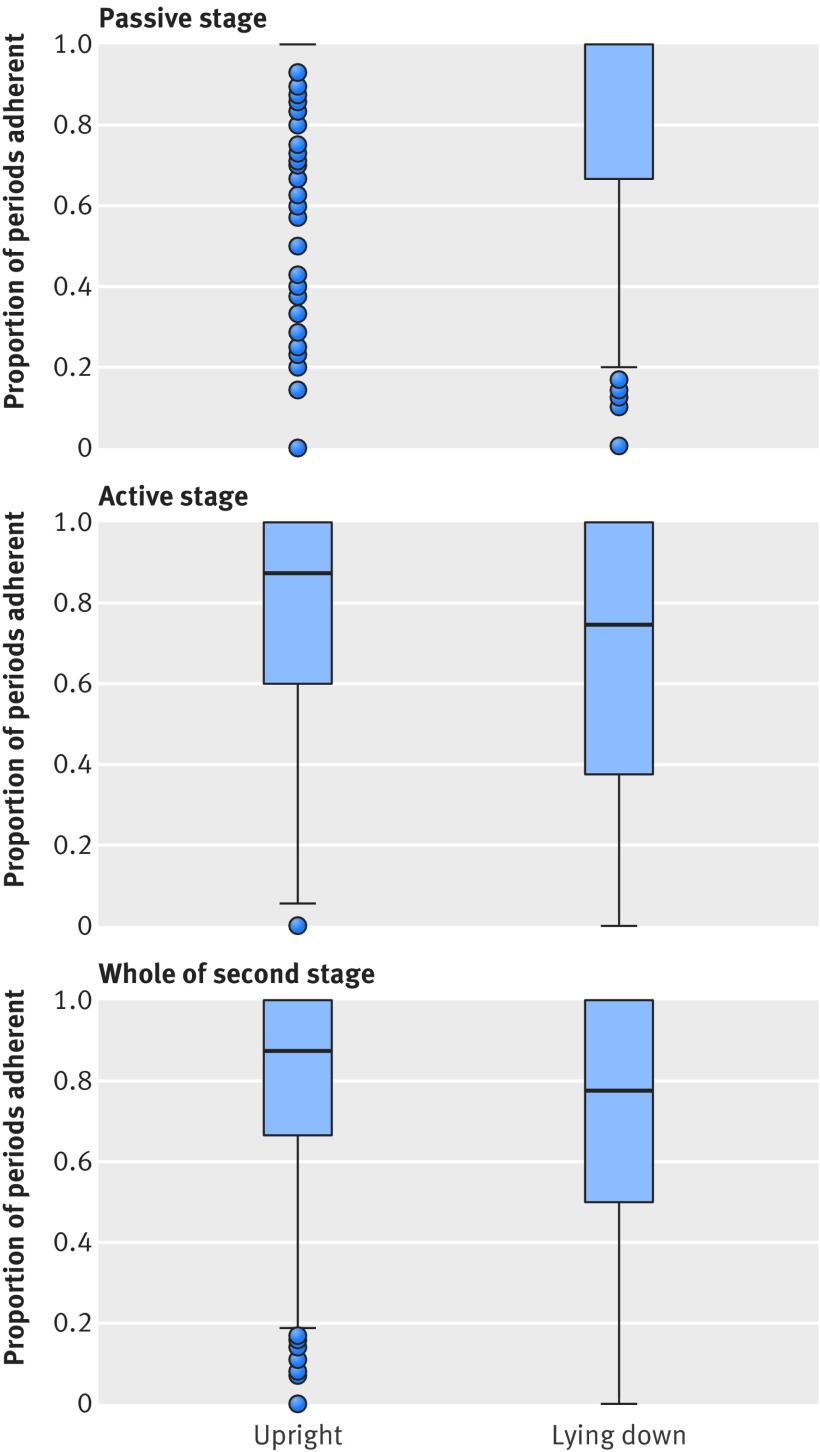
**Fig 2** Proportion of time spent in allocated position during stages of labour

A clear statistically significant difference (at the 5% level) in the incidence of the primary outcome of spontaneous vaginal birth was found between the groups, with 35.2% (548/1556) of women achieving spontaneous vaginal birth in the upright group compared with 41.1% (632/1537) in the lying down group (adjusted risk ratio 0.86, 95% confidence interval 0.78 to 0.94) (table 3). This represents a 5.9% absolute increase in the chance of spontaneous vaginal birth in the lying down group (number needed to treat 17, 95% confidence interval 11 to 40). This result was unchanged when adjusting for age, ethnicity, diagnosis of delay, and the nature of the onset of labour (adjusted risk ratio 0.86, 95% confidence interval 0.79 to 0.94).

We found no evidence of a difference for most of the secondary maternal outcomes after study entry and during the second stage of labour (tables 3 and 4[Table tbl3 tbl4]). The duration of the active second stage of labour showed a statistically significant difference at the 1% level, with a shorter duration of labour in the lying down group (median difference of 7 minutes, 99% confidence interval 0 to 13). Other secondary maternal outcomes, such as instrumental vaginal delivery, caesarean section and perineal trauma, were suggestive of an increased risk associated with the upright position, but these differences were not statistically significant at the 1% level. For example, the incidence of episiotomy increased in the upright group (58.8%, 914/1556) compared with the lying down group (54.6%, 838/1537) (although not statistically significant at the 1% level). There seemed to be a higher incidence of obstetric anal sphincter injury in the upright group (6.7%, 104/1556) compared with the lying down group (5.3%, 81/1537), but again this difference was not statistically significant at the 1% level (table 3).

**Table 3 tbl3:** Maternal primary and secondary outcomes. Values are numbers (percentages) unless stated otherwise

Outcomes	Upright (n=1556)	Lying down (n=1537)	Adjusted* effect measures
**Primary outcome**			**Effect measure (95% CI)**
Spontaneous vaginal birth	548 (35.2)	632 (41.1)	RR 0.86 (0.78 to 0.94)
**Secondary outcomes**			**Effect measure (99% CI)**
Epidural drugs† administered after study entry	832 (75.4)	862 (76.7)	
Missing	453	413	
Total dose local anaesthetic† (mg):			
Bupivacaine:	814	849	
Mean (SD)	26.4 (22.2)	26.7 (21.2)	
Median (interquartile range)	20 (10-31)	20 (12-33)	Med D 0 (−2 to 0)
Lignocaine:	6	8	
Mean (SD)	256.7 (88.0)	205 (99.6)	
Median (interquartile range)	200 (200-360)	200 (180-250)	Med D 0 (−100 to 180)
Ropivicaine:	2	1	
Mean (SD)	75 (31.8)	75	
Median (interquartile range)	75 (53-98)	75 (75-75)	Med D 0 (−23 to 23)
Total dose opioids†			
Fentanyl (µg):	809	840	
Mean (SD)	49.4 (39.0)	51.6 (41.6)	
Median (interquartile range)	40 (20-60)	40 (22-64)	Med D 0 (−4 to 0)
Diamorphine (mg):	4	1	
Mean (SD)	3.0 (0.0)	3.0	Med D 0 (0 to 0)
Median (interquartile range)	3 (3-3)	3 (3-3)	
Hypotension (systolic BP <100 mm Hg)	42 (2.7)	49 (3.2)	RR 0.85 (0.50 to 1.44)
Vasopressors to increase blood pressure	13 (0.8)	12 (0.8)	RR 1.07 (0.39 to 2.99)
Syntocinon for augmentation	172 (11.1)	163 (10.6)	RR 1.04 (0.80 to 1.35)
Fetal blood sampling performed	90 (5.8)	72 (4.7)	RR 1.17 (0.82 to 1.68)
Fetal scalp clip applied	94 (6.1)	85 (5.6)	RR 1.09 (0.76 to 1.57)
Duration of active 2nd stage§ (mins):			
Geometric mean	80.9	75.0	GMR 1.08 (1.01 to 1.15)
Median (interquartile range)	94 (56-133)	88 (51-126)	Med D 6 (1 to 11)
Total duration of 2nd stage¶ (mins):			
Geometric mean	130.5	125.1	GMR 1.04 (0.98 to 1.10)
Median (interquartile range)	149 (100-197)	141 (95-188)	Med D 7 (0 to 13)
Mode of delivery:			
Instrumental vaginal delivery**	849 (54.6)	778 (50.6)	RR 1.08 (0.99 to 1.18)
Forceps	578 (37.2)	503 (32.7)	
Ventouse	271 (17.4)	275 (17.9)	
Caesarean section††	158 (10.2)	127 (8.3)	RR 1.23 (0.92 to 1.64)
Category‡‡ of caesarean section:			
1	54 (34.2)	33 (26.0)	
2	95 (60.1)	81 (63.8)	
3	9 (5.7)	13 (10.2)	
**Primary indication for assisted (non-spontaneous) delivery**			
Instrumental:			
Fetal distress	338 (39.9)	304 (39.1)	
Failure to progress	504 (59.4)	468 (60.2)	
Other	6 (0.7)	5 (0.6)	
Caesarean section:			
Fetal distress	39 (24.7)	32 (25.2)	
Failure to progress	118 (74.7)	94 (74.0)	
Other	1 (0.6)	1 (0.8)	
Anaesthesia required for instrumental/caesarean section delivery§§	587 (58.5)	515 (57.4)	RR 1.03 (0.95 to 1.12)
Technique used¶¶:			
Local infiltration	65 (11.1)	94 (18.3)	
Pudendal block	16 (2.7)	16 (3.1)	
High dose epidural top-up	439 (74.8)	342 (66.4)	
Spinal anaesthesia	68 (11.6)	72 (14.0)	
General anaesthesia	11 (1.9)	6 (1.2)	
Active management of 3rd stage	1450 (98.0)	1432 (98.2)	RR 1.00 (0.91 to 1.10)
Missing	76	78	
**Genital tract trauma**			
Episiotomy performed	914 (58.8)	838 (54.6)	RR 1.07 (0.99 to 1.16)
Perineal tear evident (including perineal tear with episiotomy)	759 (48.9)	785 (51.1)	RR 0.95 (0.87 to 1.04)
Severity***:			
1	90 (11.9)	96 (12.2)	
2	563 (74.4)	608 (77.5)	
3a	49 (6.5)	53 (6.8)	
3b	33 (4.4)	17 (2.2)	
3c	16 (2.1)	7 (0.9)	
4	6 (0.8)	4 (0.5)	
Obstetric anal sphincter injury†††:	104 (6.7)	81 (5.3)	RR 1.27 (0.88 to 1.84)
Perineum sutured	1284 (82.6)	1248 (81.4)	RR 1.02 (0.92 to 1.13)
Anterior tear evident and sutured	102 (6.6)	107 (7.0)	RR 0.95 (0.67 to 1.33)
Manual removal of placenta	99 (6.5)	101 (6.7)	RR 0.97 (0.69 to 1.38)
Postpartum haemorrhage requiring blood transfusion:	63 (4.1)	52 (3.4)	RR 1.20 (0.75 to 1.93)
Mean (SD) units transfused‡	2.6 (1.5)	2.2 (1.0)	MD −0.34 (−0.94 to 0.27)
Woman’s pain score for birth‡‡‡:			
No	1211	1190	
Median (interquartile range)	15 (0-50)	10 (0-50)	Med D 0 (0 to 0)
Missing	345	347	
Median (interquartile range) length of inpatient stay after delivery (hrs)	38.7 (24.9-59.7)	37.5 (24.2-56.5)	Med D −1.2 (−3.2 to 0.7)
Missing	48	34	

Values for median difference (Med D) are not adjusted for centre.

BP=blood pressure; RR=risk ratio; MD=mean difference; GMR=geometric mean ratios.

Missing data are <3% unless otherwise presented. There were no apparent differences in missing data between trial groups.

*Adjusted for centre as a random effect.

†Includes “top-up” and/or patient controlled epidural anaesthesia.

‡In women who had blood transfused.

§Time from when pushing commenced until birth.

¶Time from study entry until birth.

**Compared with no instrumental vaginal delivery.

††Compared with no caesarean section.

‡‡Royal College of Obstetricians and Gynaecologists classifications: 1. immediate threat to life of woman or fetus; 2. threat of maternal or fetal compromise; 3. no threat of compromise but needs early delivery.

§§Anaesthesia additional to routine epidural pain relief given in labour.

¶¶Categories are not mutually exclusive.

***Degree of severity according to NICE intrapartum guidelines: 1. injury to skin only; 2. injury to perineal muscles but not anal sphincter; 3. injury to perineum involving anal sphincter complex (a. <50% of external anal sphincter thickness torn, b. >50% of external anal sphincter thickness torn, c. internal anal sphincter torn); 4. injury to perineum involving anal sphincter complex and anal epithelium.

†††Severity grades 3 and 4.

‡‡‡Measured using a visual analogue scale, with 0 as no pain and 10 as worst pain imaginable.

**Table 4 tbl4:** Neonatal secondary outcomes

Outcomes	Upright (n=1556)	Lying down (n=1537)	Adjusted* effect measure (99% CI)
Apgar score <4 at 5 mins	2 (0.1)	3 (0.2)	RR 0.66 (0.06 to 6.88)
Metabolic acidosis†	6 (0.4)	17 (1.2)	RR 0.35 (0.10 to 1.18)
pH and/or base deficit not done‡	531 (35.5)	597 (40.4)	
Missing	61	60	
Meconium stained liquor at delivery	347 (22.4)	341 (22.2)	RR 1.01 (0.85 to 1.19)
Resuscitation at birth	206 (13.3)	180 (11.7)	RR 1.13 (0.89 to 1.44)
Method§:			
Facial oxygen	122 (59.5)	94 (52.2)	
Suction	75 (36.6)	74 (41.1)	
Bag and mask ventilation	82 (40.0)	82 (45.6)	
Intubation	6 (2.9)	8 (4.4)	
Complex resuscitation	4 (2.0)	1 (0.6)	
Skin to skin contact in 1st hour after birth	1165 (77.1)	1163 (78.4)	RR 0.98 (0.94 to 1.03)¶
Missing	45	53	
Breast feeding initiated in 1st hour after birth	780 (51.3)	781 (52.1)	RR 0.98 (0.90 to 1.07)
Median (interquartile range) length of inpatient hospital stay (hrs) from birth	38.7 (24.8-59.7)	37.5 (24.2-56.9)	Med D** −1.1 (−3.1 to 0.8)
Missing	51	38	
Admission to higher level of care††	108 (7.0)	96 (6.3)	RR 1.11 (0.79 to 1.56)
Length of stay in higher level of care‡‡ (days)	71	63	
Median (interquartile range)	2 (1-4)	3 (1-6)	Med D** 1 (0 to 2)

RR=risk ratio; Med D=median difference.

*Adjusted for centre as a random effect.

†Cord artery pH <7.05 with base deficit ≥12 mmol/L.

‡Included in denominator.

§Categories are not mutually exclusive.

¶Unadjusted model presented as adjusted model did not converge.

**Not adjusted for centre.

††Includes transitional care.

‡‡Excludes transitional care.

Infant outcomes were good, with few babies having a low Apgar score at five minutes or evidence of metabolic acidosis. Overall, about 12% of babies required resuscitation at birth (table 4).

Maternal satisfaction in labour was similar between the two groups (table 5); however, only half of the women reported being able to move as much as they wanted. Few adverse events occurred. Mothers experienced dizziness (n=29 in the upright group and n=21 in the lying down group), postpartum haemorrhage (n=1 in each group), seizure (n=2 in lying down group), stroke (n=1 in the lying down group), maternal infection (n=1 in the upright group and n=2 in the lying down group), dural tap (n=1 in the upright group), and postpartum haemorrhage with retained placenta (n=1 in the upright group). Of infants born to mothers in the upright group one was a stillbirth, one experienced birth trauma, one had cardiorespiratory collapse one hour post birth, and one had suspected Erb’s palsy.

**Table 5 tbl5:** Maternal satisfaction. Values are numbers (percentages) unless stated otherwise

Outcomes	Upright (n=1556)	Lying down (n=1537)
No of questionnaires returned	1208 (77.6)	1165 (75.8)
Satisfied with overall childbirth experience:		
Strongly agree	553 (47.2)	539 (47.1)
Agree	410 (35.0)	434 (37.9)
Neutral	114 (9.7)	100 (8.7)
Disagree	65 (5.6)	40 (3.5)
Strongly disagree	30 (2.6)	31 (2.7)
Treated with respect by all staff:		
Strongly agree	968 (82.0)	937 (81.3)
Agree	178 (15.1)	176 (15.3)
Neutral	19 (1.6)	20 (1.7)
Disagree	7 (0.6)	11 (1.0)
Strongly disagree	8 (0.7)	8 (0.7)
Involved in making decisions:		
Strongly agree	824 (69.9)	788 (68.5)
Agree	278 (23.6)	299 (26.0)
Neutral	56 (4.8)	45 (3.9)
Disagree	11 (0.9)	10 (0.9)
Strongly disagree	10 (0.9)	9 (0.8)
Expectations for labour & birth were met:		
Strongly agree	444 (38.0)	437 (38.2)
Agree	359 (30.7)	346 (30.2)
Neutral	209 (17.9)	207 (18.1)
Disagree	118 (10.1)	113 (9.9)
Strongly disagree	40 (3.4)	41 (3.6)
Felt safe at all times:		
Strongly agree	793 (67.4)	773 (67.2)
Agree	312 (26.5)	299 (26.0)
Neutral	39 (3.3)	51 (4.4)
Disagree	24 (2.0)	16 (1.4)
Strongly disagree	9 (0.8)	11 (1.0)
Good communication from staff:		
Strongly agree	913 (77.3)	864 (75.3)
Agree	222 (18.8)	230 (20.0)
Neutral	30 (2.5)	33 (2.9)
Disagree	9 (0.8)	10 (0.9)
Strongly disagree	7 (0.6)	11 (1.0)
Felt in control:		
Strongly agree	428 (36.4)	426 (37.2)
Agree	396 (33.6)	368 (32.1)
Neutral	223 (19.0)	232 (20.2)
Disagree	105 (8.9)	93 (8.1)
Strongly disagree	25 (2.1)	27 (2.4)
Able to move as much as wanted:		
Strongly agree	283 (24.5)	310 (27.2)
Agree	285 (24.7)	279 (24.5)
Neutral	239 (20.7)	236 (20.7)
Disagree	253 (21.9)	228 (20.0)
Strongly disagree	95 (8.2)	86 (7.6)
Missing	53	26
Satisfied with position before pushing:		
Strongly agree	590 (50.3)	566 (49.4)
Agree	460 (39.2)	430 (37.5)
Neutral	83 (7.1)	83 (7.2)
Disagree	29 (2.5)	52 (4.5)
Strongly disagree	12 (1.0)	15 (1.3)
Satisfied with position while pushing:		
Strongly agree	613 (52.2)	570 (49.8)
Agree	425 (36.2)	422 (36.9)
Neutral	94 (8.0)	91 (8.0)
Disagree	29 (2.5)	48 (4.2)
Strongly disagree	13 (1.1)	14 (1.2)
Satisfied with labour pain relief:		
Strongly agree	791 (67.2)	774 (67.4)
Agree	300 (25.5)	288 (25.1)
Neutral	60 (5.1)	51 (4.4)
Disagree	14 (1.2)	23 (2.0)
Strongly disagree	12 (1.0)	13 (1.1)
Missing	31	16

The prespecified subgroup analyses showed no evidence of heterogeneity between any of the prespecified subgroups for the primary outcome of spontaneous vaginal birth (fig 3).

**Figure f3:**
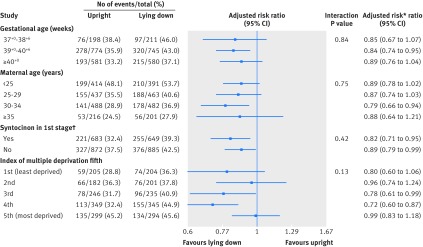
**Fig 3** Forest plot showing results of subgroup analyses for spontaneous vaginal birth. *All models adjust for centre as a random effect. †Diagnosis of delay before study entry requiring syntocinon

No evidence of any differences between the groups was found for incidence or severity of urinary incontinence, faecal incontinence, constipation, haemorrhoids or dyspareunia, general wellbeing, or health related quality of life (table 6). This finding was unchanged in a sensitivity analysis where women were excluded if they were pregnant or had another child by the time of the one year follow-up (table 7). Similarly, there was no evidence of a difference in the incidence of diagnosed cerebral palsy or severe neurodevelopmental delay in any of the infants at one year: major morbidity was experienced by 0.1% (1/950) in the upright group and 0.4% (4/942) in the lying down group, with a risk ratio adjusted for centre as a random effect of 0.25 (99% confidence interval 0.01 to 4.40). The response rate to the one year follow-up was 61.2% (1892/3093). There were differences between women who responded and those who did not respond, with responders tending to be slightly older, white, and from less deprived areas, and more likely to have an instrumental vaginal birth with evidence of perineal trauma. However, there were no apparent differences in the two randomised groups in their response rates or characteristics, suggesting no major biases in the comparison between the two groups (table 8).

**Table 6 tbl6:** Secondary maternal and infant outcomes up to one year. Values are numbers (percentages) unless stated otherwise

Outcomes	Upright (n=950)	Lying down (n=942)	Adjusted* effect measure (99% CI)
**Urinary incontinence**			
Leakage in first 3 months	462 (48.8)	461 (49.2)	RR 0.99 (0.88 to 1.12)
ICIQ-UI score† in past 4 weeks:			
Median (interquartile range) score	0 (0-4)	0 (0-4)	Med D‡ 0 (0 to 0)
Missing	39	33	
**Faecal incontinence**			
No bowel control and/or soiling:			
In first 3 months	108 (11.5)	132 (14.2)	RR 0.81 (0.59 to 1.11)
In past 4 weeks	32 (3.4)	27 (2.9)	RR 1.18 (0.61 to 2.28)
No bowel control and/or soiling and/or feel need to go:			
In first 3 months	215 (22.8)	251 (26.9)	RR 0.85 (0.69 to 1.05)
In past 4 weeks	113 (12.1)	102 (10.9)	RR 1.10 (0.79 to 1.53)
No bowel control at times§:			
Never	829 (87.9)	806 (86.0)	
In first 3 months	83 (8.8)	103 (11.0)	
In past 4 weeks	13 (1.4)	19 (2.0)	
At any other time	29 (3.1)	20 (2.1)	
Soiling on underwear§:			
Never	836 (88.6)	838 (89.5)	
In first 3 months	70 (7.4)	75 (8.0)	
In past 4 weeks	24 (2.5)	14 (1.5)	
At any other time	24 (2.5)	22 (2.4)	

Feel need and have to go immediately§:			
Never	640 (67.9)	616 (65.8)	
In first 3 months	173 (18.4)	202 (21.6)	RR 0.85 (0.67 to 1.08)
In past 4 weeks	98 (10.4)	90 (9.6)	RR 1.08 (0.76 to 1.55)
At any other time	77 (8.2)	82 (8.8)	
Constipation§:			
Never	367 (38.9)	406 (43.2)	
In first 3 months	395 (41.8)	353 (37.6)	RR 1.12 (0.96 to 1.29)
In past 4 weeks	94 (10.0)	107 (11.4)	RR 0.87 (0.62 to 1.23)
At any other time	140 (14.8)	154 (16.4)	
Haemorrhoids§:			
Never	495 (52.4)	518 (55.1)	
In first 3 months	308 (32.6)	297 (31.6)	RR 1.03 (0.87 to 1.23)
In past 4 weeks	108 (11.4)	116 (12.3)	RR 0.93 (0.67 to 1.28)
At any other time	108 (11.4)	115 (12.2)	
Dyspareunia§¶:			
Never	366 (40.7)	363 (40.6)	
In first 3 months	364 (40.5)	381 (42.6)	RR 0.95 (0.82 to 1.10)
In past 4 weeks	80 (8.9)	79 (8.8)	RR 1.01 (0.68 to 1.49)
At any other time	160 (17.8)	151 (16.9)	
Not applicable (not had sexual intercourse since the birth)	46	45	
**Health related quality of life**			
Mean (SD) EQ-5D-3L score	0.922 (0.150)	0.922 (0.138)	MD −0.0004 (−0.014 to 0.013)
Missing EQ-5D-3L score	102	100	
Mean (SD) SF-6D	0.802 (0.120)	0.805 (0.120)	MD −0.003 (−0.014 to 0.008)
Major infant morbidity**	1 (0.11)	4 (0.42)	RR 0.25 (0.01 to 4.40)

RR=risk ratio; Med D=median difference; MD=mean difference.

Missing data are <3% unless otherwise presented. There were no apparent differences in missing data between trial groups.

*Adjusted for centre as a random effect.

†International consultation on incontinence questionnaire–urinary incontinence form (scale from 0 to 21, a high score indicating worse problems).

‡Not adjusted for centre.

§Woman could tick more than one option so percentages may total >100%.

¶Excludes women who have not had sexual intercourse.

**For example, gross neurodevelopmental delay, including cerebral palsy.

**Table 7 tbl7:** Sensitivity analysis: one year maternal outcomes excluding women who had another child or were pregnant at time of assessment. Values are numbers (percentages) unless stated otherwise

Outcomes	Upright (n=950)	Lying down (n=942)	Adjusted^*^ effect measure (99% CI)
Women who have had another baby	6 (0.6)	4 (0.4)	
Women pregnant at time of completing questionnaire	61 (6.5)	72 (7.8)	
Denominator excluding women pregnant/had another baby	883	866	
**Urinary incontinence**			
Leakage in first 3 months	432 (49.2)	426 (49.4)	RR 0.99 (0.88 to 1.13)
ICIQ-UI score† over past 4 weeks:			
Median (interquartile range)	0 (0-4)	0 (0-4)	Med D‡ 0 (0 to 0)
Missing	38	30	
**Faecal incontinence**			
No bowel control and/or soiling:			
In first 3 months	101 (11.5)	122 (14.2)	RR 0.81 (0.59 to 1.12)
In past 4 weeks	28 (3.2)	27 (3.2)	RR 1.02 (0.51 to 2.02)
No bowel control and/or soiling and/or feel need to go:			
In first 3 months	203 (23.2)	235 (27.4)	RR 0.85 (0.68 to 1.05)
In past 4 weeks	106 (12.2)	93 (10.9)	RR 1.12 (0.79 to 1.58)
Feel need to go and have to go immediately§:			
In first 3 months	161 (18.4)	191 (22.2)	RR 0.83 (0.65 to 1.06)
In past 4 weeks	92 (10.5)	81 (9.4)	RR 1.12 (0.77 to 1.62)
Constipation§:			
In first 3 months	368 (42.0)	328 (38.0)	RR 1.11 (0.95 to 1.29)
In past 4 weeks	82 (9.4)	90 (10.4)	RR 0.90 (0.62 to 1.30)
Haemorrhoids§:			
In first 3 months	291 (33.2)	278 (32.2)	RR 1.03 (0.86 to 1.23)
In past 4 weeks	100 (11.4)	102 (11.8)	RR 0.97 (0.69 to 1.36)
Dyspareunia§¶:			
In first 3 months	339 (40.7)	351 (42.9)	RR 0.95 (0.82 to 1.10)
In past 4 weeks	75 (9.0)	78 (9.5)	RR 0.95 (0.64 to 1.41)
Not applicable (not had sexual intercourse since the birth)	45	45	

RR=risk ratio; Med D=median difference.

Missing data are <3% unless otherwise presented. There were no apparent differences in missing data between trial groups.

*Adjusted for centre as a random effect.

†International consultation on incontinence questionnaire–urinary incontinence form (scale from 0 to 21, a high score indicating worse problems).

‡Not adjusted for centre.

§Woman could tick more than one option so percentages may total >100%.

¶Excludes women who have not had sexual intercourse.

**Table 8 tbl8:** Generalisability of women followed-up. Values are numbers (percentages) unless stated otherwise

Characteristics	1 year follow-up			P value	
	Received (n=1892)	Not received (n=1201)		Received *v* not received	Interaction with allocation*
Mean (SD) maternal age (years)	29.7 (5.2)	26.5 (5.7)		<0.001†	0.76
Mean (SD) gestational age at entry (weeks)	40.4 (1.2)	40.3 (1.2)		0.05†	0.08
Index of multiple deprivation (fifth):				<0.001‡	0.86
First (least deprived)	279 (17.6)	130 (13.3)			
Second	259 (16.4)	124 (12.7)			
Third	318 (20.1)	163 (16.7)			
Fourth	431 (27.2)	263 (26.9)			
Fifth (most deprived)	295 (18.7)	298 (30.5)			
Wales: not derived	265	176			
Postcode missing	45	47			
Ethnic group:				<0.001‡	0.95
White	1624 (86.5)	956 (80.1)			
Indian	58 (3.1)	47 (3.9)			
Pakistani	22 (1.2)	34 (2.9)			
Bangladeshi	3 (0.2)	6 (0.5)			
Black African	30 (1.6)	28 (2.4)			
Black Caribbean	11 (0.6)	14 (1.2)			
Other	129 (6.9)	109 (9.1)			
Mean (SD) body mass index (at booking visit):					
Mean (SD)	25.2 (5.2)	25.6 (5.6)		0.03†	0.65
Height and/or weight not known	70	55			
Onset of labour:					
Spontaneous	1121 (59.3)	724 (60.3)		0.57‡	0.89
Induced	769 (40.7)	476 (39.7)			
Diagnosis of delay requiring intervention	985 (52.1)	581 (48.4)		0.04‡	0.18
Spontaneous vaginal birth	677 (35.8)	503 (41.9)		0.001‡	0.73
Instrumental vaginal delivery§	1040 (55.0)	587 (48.9)		0.001‡	0.88
Caesarean section¶	175 (9.3)	110 (9.2)		0.94‡	0.63
Episiotomy performed	1120 (59.2)	632 (52.7)		<0.001‡	0.61
Obstetric anal sphincter injury**	116 (6.1)	69 (5.8)		0.68‡	0.37
Perineum sutured	1585 (83.9)	947 (79.1)		0.001‡	0.54
Resuscitation at birth	241 (12.8)	145 (12.1)		0.58‡	0.22
Breast feeding initiated in first hour after birth	994 (53.8)	567 (48.4)		0.004‡	0.11
Missing	45	29			
Infant admission to higher level of care††	121 (6.4)	83 (6.9)		0.57‡	0.76

Missing data are <3% unless otherwise presented. There were no apparent differences in missing data between trial groups.

*Logistic regression analysis performed predicting follow-up received.

†Student’s *t*-test performed.

‡χ^2^ test performed.

§Compared with no instrumental vaginal delivery.

¶Compared with no caesarean section.

**Severity grades 3 and 4.

††Includes transitional care.

## Discussion

Evidence from this randomised controlled trial indicates that a policy of adopting a lying down position in the second stage of labour in women having their first baby with epidural analgesia increases the chances of a spontaneous vaginal birth (number needed to treat 17 women (95% confidence interval 11 to 40) to achieve one additional spontaneous vaginal birth). No disadvantages were apparent to short or longer term outcomes for mother or baby.

### Strengths and limitations of this study

As with all pragmatic trials, this study has limitations. The incidence of spontaneous vaginal birth in this population of women was lower than anticipated. Our original sample size calculation was based on a spontaneous vaginal birth rate of approximately 55% in the control group.^4^ This trend towards higher rates of intervention in the second stage of labour—both instrumental delivery and caesarean section—has been previously noted.^15^ Instrumental delivery is associated with high rates of perineal trauma and subsequent morbidity, particularly faecal incontinence.

With an intervention such as this, masking is impossible, so the results may be influenced by the women’s and the midwives’ perceptions of the different positions in their ability to achieve a spontaneous vaginal birth. Given that existing guidance from the National Institute for Health and Care Excellence recommends that women with an epidural should be encouraged to adopt whatever upright position they find comfortable, it is perhaps not surprising that adherence was lower in the lying down group than in the upright group, causing a possible dilution of the treatment effect.^16^ Owing to the unmasked nature of the intervention and the possibility that some midwives may firmly believe that the upright position is preferable, we might also expect the trial results to suggest an improvement in spontaneous vaginal birth with an upright position. The finding that the lying down position increased the chances of achieving a spontaneous vaginal birth suggests that this potential bias was either absent or minimal in its impact, or that the benefit of the lying down position may be even greater in leading to a spontaneous vaginal birth.

We can only speculate about the mechanism by which lying down increases the chance of a spontaneous vaginal birth in nulliparous women with a low dose epidural. We have no direct measurements of the density of the epidural block in the two positions nor the level of the block as second stage progressed. It is possible that women in the upright position acquired a denser block around the birth canal resulting from the potential effects of posture and gravity on the spread of drugs within the epidural space, which could in turn have made expulsive efforts less effective. However, the similarity of drug doses throughout the second stage of labour used in each group would suggest that this is unlikely. Women in the upright group, who may have been sitting, might have had a restricted pelvic outlet because of pressure on their coccyx or because of venous obstruction causing lower genital tract oedema and obstruction of the soft tissues of the pelvic outlet. In addition, it is possible that the lying down group, by easing pressure of the fetal head on the pelvis had improved uterine blood flow and therefore improved uterine activity. This would suggest a difference in the risk of operative delivery associated with failure to progress. The distribution of indications for operative delivery, however, appeared to be the same in either group. In addition, little difference was found in the use of oxytocin because of delay in labour progress after trial entry.

There was a suggestion that perineal trauma might also have been decreased in the lying down group, although differences in these inter-related outcomes were not statistically significant at our prespecified 1% level. A recent large observational study from Sweden, using routine data, found a statistically significant lower incidence of obstetric anal sphincter injury in women in the lateral position compared with the sitting group (risk ratio 0.79, 95% confidence interval 0.68 to 0.92).^17^ This result is similar to our finding (adjusted risk ratio 0.79, 95% confidence interval 0.59 to 1.04). Given that an increase in spontaneous vaginal births results in a decrease in operative births, it would follow that perineal trauma would be increased in the group with more instrumental deliveries. There is also a suggestion (not statistically significant at the 1% level) that instrumental vaginal delivery might be increased in the upright group in this trial.

In this trial the lack of any difference on longer term outcomes such as faecal incontinence is of interest. The existing observation that instrumental vaginal delivery is associated with an increased risk of faecal incontinence is robust.^18^ ^19^ ^20^ ^21^ The likely explanation in this trial is that the difference between the randomised groups of women in their risk of spontaneous vaginal birth was relatively small, meaning that the impact of different modes of birth on long term outcomes is diluted.

### Comparison with other studies

The existing evidence from randomised controlled trials is inconclusive. The Cochrane review^7^ contains five trials including a total of 879 women, with clinical heterogeneity between the trials. For example, some women were actively encouraged to walk, and others were supported in a sitting position in the upright group. In the recumbent group, some trials had women sitting and others had women in a lateral position. This trial adds a further 3000 women to this evidence, with clearly defined comparison groups. When these data are added to the three most comparable trials—that is, those where women were allocated upright versus lying down positions (as opposed to semi-recumbent positions), then the sum of the evidence strongly supports a lying down position to achieve a spontaneous vaginal birth (upright versus lying down meta-analysis odds ratio 0.80, 95% confidence interval 0.70 to 0.92).

### Conclusions and policy implications

This study provides evidence that adopting a lying down position in the second stage of labour results in more spontaneous vaginal births in nulliparous women with epidural analgesia, with no apparent disadvantages for short or longer term outcomes for mother or baby. The intervention seems to be easy to adopt and is cost free. This evidence will allow pregnant women, in consultation with their healthcare providers, to make informed choices about their position in the second stage of labour.

The results from this trial apply to nulliparous women in the second stage of labour with epidural analgesia. It is unclear what the findings mean for multiparous women in labour with an epidural. However, women should be offered the choice of adopting a lying down position in the second stage until proven otherwise. We also do not know what these results mean for women of all parities without an epidural. Given the variable quality of existing randomised trials of position in the second stage of labour in women without an epidural,^6^ the results of the BUMPES trial strongly support the development of a similar large pragmatic trial with clear operational descriptions of position in women in labour without epidural analgesia.

**What is already know on this topic**

Women who use an epidural for pain relief in labour are more likely to have an instrumental vaginal birth than those who use other methods of analgesia
Maternal position in the second stage of labour (after the cervix is fully dilated) may affect the incidence of spontaneous vaginal birth, but the existing evidence from randomised controlled trials is unclear

**What this study adds**

In nulliparous women in labour at term with an epidural and a singleton fetus, a policy of adopting a lying down position (left or right lateral) during the second stage of labour increases the chance of spontaneous vaginal birth compared with a policy of adopting an upright position
There were no adverse consequences of this approach for mother or baby in the short term, or at 12 months post birth

## Web extra

Extra material supplied by authors

**Supplementary information:** Participant centres and recruitment

**Supplementary information:** Details of trial groups, committees, staff, and recruiting centres

## Notes

We thank the women who agreed to join the trial. Writing committee: Debra Bick, professor of evidence based midwifery practice, Kings College London; Annette Briley, consultant midwife, Guys and St Thomas’s NHS Foundation Trust; Peter Brocklehurst, professor of women’s health, UCL, and professor of women’s health, director of Birmingham Clinical Trials Unit, University of Birmingham; Pollyanna Hardy, senior trials statistician, University of Oxford; Edmund Juszczak, director, NPEU Clinical Trials Unit, University of Oxford; Lynn Lynch, midwifery lecturer, Cardiff University; Christine MacArthur, professor of maternal and child epidemiology, University of Birmingham; Phillip Moore, consultant anaesthetist, University Hospital Birmingham NHS Trust; Mary Nolan, professor of perinatal education, University of Worcester; Oliver Rivero-Arias, senior health economist, University of Oxford; Julia Sanders, consultant midwife, Cardiff University, and reader in midwifery, Cardiff & Vale Health Board; Andrew Shennan, professor of obstetrics, Kings College London; Matt Wilson, consultant in obstetric anaesthesia, Sheffield Teaching Hospital, and senior lecturer in anaesthesia, University of Sheffield.

Ethical approval: This study was approved by the National Research Ethics Service—Oxfordshire research ethics committee B (reference No 09/H0605/114). The study protocol can be accessed at www.ucl.ac.uk/cctu/researchareas/womenshealth/bumpes/documents/protocolv5.

Data sharing: Requests for access to data from the BUMPES trial should be addressed to the corresponding author.

Transparency: The lead author (PB) affirms that this manuscript is an honest, accurate, and transparent account of the study being reported; that no important aspects of the study have been omitted; and that any discrepancies from the study as planned (and, if relevant, registered) have been explained.
