# Supplementary material for: Upright versus lying down position in second stage of labour in nulliparous women with low dose epidural: BUMPES randomised controlled trial
Source: BMJ. 2017 Oct 18;359:j4471. doi: 10.1136/bmj.j4471 (PMC5646262; doi:10.1136/bmj.j4471)
Supplement: Supplementary file 1 — Supplementary information: Participant centres and recruitment [file bird039017.wt1.pdf]

Supplementary table Participation centres and recruitment

Characteristic	Upright (n=1556)		Lying down (n=1537)	
	n	(%)	n	(%)
Centre				
Birmingham Women's Hospital	116	(7.5)	118	(7.7)
St Thomas' Hospital, London	237	(15.2)	241	(15.7)
Queen Alexandra Hospital, Portsmouth	43	(2.8)	42	(2.7)
University Hospital of Wales, Cardiff	150	(9.6)	146	(9.5)
Royal United Hospital, Bath	99	(6.4)	101	(6.6)
Bradford Royal Infirmary Bradford	58	(3.7)	55	(3.6)
Jessop Wing, Sheffield	93	(6.0)	94	(6.1)
Princess of Wales Hospital, Bridgend	22	(1.4)	19	(1.2)
Singleton Hospital, Swansea	19	(1.2)	18	(1.2)
Royal Gwent Hospital, Newport	29	(1.9)	25	(1.6)
Gloucestershire Royal Hospital	26	(1.7)	22	(1.4)
Nevill Hall Hospital, Abergavenny	9	(0.6)	10	(0.7)
Frimley Park Hospital, Camberley	97	(6.2)	96	(6.3)
Sunderland Royal Hospital	21	(1.4)	22	(1.4)
Pinderfields Hospital, Wakefield	36	(2.3)	36	(2.3)
Warrington Hospital	29	(1.9)	29	(1.9)
Tameside Hospital, Ashton-under-Lyne	26	(1.7)	24	(1.6)
Medway Maritime Hospital, Gillingham	15	(1.0)	10	(0.7)
South Tyneside District Hospital, South Shields	8	(0.5)	7	(0.5)
Queen Mary's Hospital, Sidcup	64	(4.1)	62	(4.0)
Queen Charlotte's & Chelsea Hospital, London	7	(0.5)	11	(0.7)
Queen Elizabeth Hospital, Birmingham	24	(1.5)	21	(1.4)
Great Western Hospital, Swindon	27	(1.7)	30	(2.0)
Royal Cornwall Hospital, Truro	19	(1.2)	20	(1.3)
Bedford Hospital Bedford	26	(1.7)	30	(2.0)
University College Hospital, London	18	(1.2)	13	(0.9)
Royal Sussex County Hospital, Brighton	16	(1.0)	13	(0.9)
North Manchester General Hospital	30	(1.9)	28	(1.8)
New Cross Hospital. Wolverhampton	22	(1.4)	19	(1.2)
James Paget University Hospital, Great Yarmouth	21	(1.4)	23	(1.5)
St George's Hospital, London	32	(2.1)	33	(2.2)
Princess Royal Hospital, Glasgow	6	(0.4)	2	(0.1)
King's College London	39	(2.5)	41	(2.7)
St Mary's Hospital, London	3	(0.2)	4	(0.3)
Dorset County Hospital, Dorchester	10	(0.6)	10	(0.7)
Kingston Hospital	41	(2.6)	46	(3.0)
Hillingdon Hospital	7	(0.5)	5	(0.3)
Arrowe Park Hospital, Wirral	7	(0.5)	5	(0.3)
Lewisham Hospital, London	2	(0.1)	1	(0.1)
Prince Charles Hospital, Merthyr Tydfil	2	(0.1)	5	(0.3)
