# Supplementary material for: Upright versus lying down position in second stage of labour in nulliparous women with low dose epidural: BUMPES randomised controlled trial
Source: BMJ. 2017 Oct 18;359:j4471. doi: 10.1136/bmj.j4471 (PMC5646262; doi:10.1136/bmj.j4471)
Supplement: Supplementary file 2 — Supplementary information: Details of trial groups, committees, staff, and recruiting centres [file bird039017.ww1.pdf]

Investigator group:

- Professor Debra Bick, Professor of Evidence Based Midwifery Practice, Kings College London
- Dr Annette Briley, Consultant Midwife, Guys and St Thomas's NHS Foundation Trust (replaced Geraldine O'Sullivan, Lead Clinician in Obstetric Anaesthesia in 2012)
- Professor Peter Brocklehurst, Director, Institute for Women's Health, UCL; Director Birmingham Clinical Trials Unit, University of Birmingham
- Oya Eddama, Health Economist, National Perinatal Epidemiology Unit, Oxford (until September 2015)
- Professor Janesh Gupta, Professor/Honorary Consultant in Obstetrics and Gynaecology, Birmingham University/Birmingham Women's Foundation NHS Trust
- Pollyanna Hardy, Senior Trials Statistician, National Perinatal Epidemiology Unit Clinical Trials Unit, University of Oxford
- Associate Professor Edmund Juszczak, Director, National Perinatal Epidemiology Unit Clinical Trials Unit, University of Oxford
- Lynn Lynch, Trial Senior Research Midwife
- Professor Christine MacArthur, Professor of Maternal and Child Epidemiology, University of Birmingham
- Professor Rona McCandlish, Epidemiologist: Maternal Health, National Perinatal Epidemiology Unit, Oxford (until 2012)
- Dr Phillip Moore, Consultant Anaesthetist, University Hospital Birmingham NHS Trust
- Professor Mary Nolan, Professor of Perinatal Education, University of Worcester
- Dr Geraldine O'Sullivan, Lead Clinician in Obstetric Anaesthesia, Guys and St Thomas's NHS Foundation Trust (deceased 2012)
- Dr Felicity Plaat, Lead Clinician & Consultant Anaesthetist, Queen Charlotte's and the Hammersmith Hospital/ Senior Lecturer, Imperial College London
- Dr Dean Regier, Senior Health Economist, National Perinatal Epidemiology Unit, Oxford (until 2012)
- Dr Julia Sanders, Consultant Midwife/Reader in Midwifery, Cardiff University
- Professor Andrew Shennan, Professor of Obstetrics, Kings College London

- Dr Matt Wilson, Consultant in Obstetric Anaesthesia / Senior Lecturer in Anaesthesia, Sheffield Teaching Hospital/University of Sheffield
- Associate Professor Oliver Rivero-Arias, Senior Health Economist, National Perinatal Epidemiology Unit, University of Oxford

Trial steering committee:

- Dr Paul Howell, Consultant Anaesthetist, St Bartholomew's Hospital
- Professor Dame Tina Lavender, Professor in Midwifery, University of Manchester
- Professor Alan Montgomery (Vice-Chair), Professor of Medical Statistics and Clinical Trials, Nottingham Clinical Trials Unit
- Professor Stephen Palmer, Professor of Health Economics, University of York
- Ms Justine Pepperell (Consumer Representative)
- Professor Steve Robson (Chair), Professor of Fetal Medicine, Medical School, University of Newcastle

Data monitoring committee

- Professor Christine Kettle, Professor of Women's Health, Academic Unit of Obstetrics and Gynaecology, University Hospital of North Staffordshire and Staffordshire University
- Mr Stephen Walkinshaw (Vice-Chair), Consultant in Maternal and Fetal Medicine, Liverpool Women's NHS Foundation Trust
- Dr Steve Yentis (Chair), Consultant Anaesthetist, Chelsea & Westminster Hospital
- Dr Pat Yudkin, Emeritus Reader in Medical Statistics, University of Oxford

Clinical trials unit staff:

National Perinatal Epidemiology Unit, University of Oxford (1.1.10 to 31.8.2011):

- Ursula Bowler, Senior Trials Manager (until August 2011)
- Sonali De Silva-Mitter, Trial Manager (until August 2011)
- Oya Eddama, Health Economist (until September 2015)
- Ann Kennedy, Assistant Trials Manager (until August 2011)

- Andy King, Head of Trials Programming (until August 2011)
- Andy Kirk, Webmaster & Design Coordinator (until August 2011)
- Claire Nelis, Statistician (until August 2011)
- Rachel Roberts, Trial Manager (until August 2011)
- Suzanne Williams, Data Coordinator (until August 2011)

Comprehensive Clinical Trials Unit, UCL (1.9.2011 to 30.9.2015):

- Julie Bakobaki, Clinical Project Manager (until April 2014)
- Laura Custins, Trial Manager (until November 2012)
- Suzanne Drake, Data Entry Assistant (until June 2015)
- Amber Gibney, Data Manager (until October 2013)
- Steve Hibbert, IT Manager (until September 2015)
- Elizabeth Howden, Trial Manager, (until December 2013)
- Alycia Kopec, Data Manager (until 2012)
- Tola Lawal, Data Manger (until January 2015)
- Sawretse Leslie, Trial Manager (until September 2015)
- Lynn Lynch, Senior Research Midwife (until June 2014)
- Kate Maclagan, Clinical Project Manager (until September 2015)
- Garrie Powers, IT Developer (until September 2015)
- Guy Schroeter, Clinical Project Manager (until April 2015)

Recruiting Centres:

Arrowe Park Hospital, Wirral: Suresh Singaravelu (Principal Investigator), Carly Nulty, Carolyn Bragg, Gerri Griffiths, Helen Burghall, Jane Murphy, Julie Dale, K. Mccoy, Lynne Lacy, Rachel Roberts, Sandra Hutcheon, Tanya Wynne, Tracy Green, Vickie Heller; *Bedford Hospital:* Yaqub Latoo (Principal Investigator), Anita Males, Anni Price, Babs Harris, C. Dyer, Carla Ball, Carol Handrahan, Donna James, Elizabeth Carlyle, Emma Clarke, Hayley Smith, Jen Welsh, Jenny Cowland, K. Emery, Katie Summers, L. Church, Liz Dodd, Lucy Wills, Marion Moore, Melissa Coles, Paula Griffiths, Rachel Pressley, Rebecca Adcock, Ruth Croot, Ruth Steward, S. Harris, Sarah Gates, Sarah Johnson-Clarke, Sue Hill; *Birmingham Women's Hospital:* Phil Moore (Principal Investigator), A. Connolly, Alexandra Bellamy, Anna Zhao, Anya Chruscinska, B. Oniono Kuafor, Becky Cullen, Bethany Lunn, Bobby Sharma, C. Bishop, C. Graves, Charlotte Bowman, Charlotte Davies, Charlotte Hinks, Chloe O'Hara, Claire Bissell,

Debbie Baker, Deborah Robinson, Elisha Randell, Elizabeth Ewers, Emily Byrne, Emma Wright, Erica Henry, Fiona Musgrave, G. George, Gemma Barnfield, Gemma Wadsworth, Hannah Wood, Harriet Fisher, J. Rose, Jane McNair, Jennifer Prescot, Jenny Pledger, Jess Shaw, Jessica Gregory, Joelle Rowland, Juillete Webster, Julia Cheshire, Justine Craig, K. Horton, Karen Davies, Karen Elkin, Katie Freitas, Kerry Hudson, Kimberley McMahon, Kiranjit Sehmi, Kirsten Emson, Kirsty Elwell, Laura Mulryan, Lauren Hill, Lauren Webley, Lisa Salmon, Lucy O'Grady, Madeleine Parry, Margarita Bariou, Maureen Joseph, Megan Corbett, Michaela Dzioba, Michaelene Cole, Michelle Bennett, Michelle Neal, Nichol Ross, Nicola Mcenery, Nikki Robbins, Novia Samuels, Orphelia Atkins, Pam Simpson, Paula Trinham, Rachel Singer, Rebecca Cullen, Rebecca Gallimore, Rebecca Leon, Rebecca Mckenzie, Rhea Bond, Ruth Cavey-Wilcox, Samuel Todd, Sarah Blythe, Sarah Ketley, Sasha Hamilton, Sethenia Beckford, Sian Wilkie, Stella Bibb, Stephanie Henry, Teresa Vann, Tracey Bond, V. Preece, Victoria McDonagh; *Bradford Royal Infirmary:* Diane Farrar (Principal Investigator), Alexandra Fozard, Alice Tunney, Alison Chapman, Amanda Wilson-Thompson, Aongola Ngenda, Carrie Owens, Christina Scott, Clare Cummings, Fozia Arshad, Geraldine Atkinson, Gillian Butterfield, Heather Darlow, Helen M Sharp, Jennie Robertshaw, Joanne Mortimer, Joanne Watson, Josephine Hartley, Judith Lowther, Katarzyna Denkiewicz, L. Jarockyj, Laura Mckenna, Lisa Thompson, Lydia Brookes, Maryanne Naylor, Nicola Davies, Rachael Jones, Rachel Hemers, Rachel Wild, Rebecca Skelton, S. Marriott, S. Nicholson, Sania Iqbal, Stacey Ryles, Susie Weekes, Talitha Grandison, Tracey Germaine-Rylance, Victoria Jones; *Brighton Royal Sussex County Hospital:* Vanessa Fludder (Principal Investigator), Claire Miles, Emma May, Florence Crawley, Hannah Tomms, Igone Sesma, Jayne Denyer, Kate McCambridge, Melanie Wight, Nichola Tuck, Omotoyin Awonuga, Paula Alonso-Gonzalez, Rachael Chatterton, Rachel Cox, Rosie Darling, Victoria Wright; *Dorset County Hospital, Dorchester:* Christine Grother (Principal Investigator), Allison Hamilton, Bev Robertson, Carly Smith, Christine Grother, Hilary Fletcher, Jane Linger, Jo Hartley, Julie Bonifacio, Julie Younger, Karen Myers, Kathryn Dyer, Louise Shreiber, Nichola Coliandris, Sarah Haigh, Tina Parker; *Frimley Park Hospital:* Karen Plews (Principal Investigator), Abbey Ford, Alexandra Mayrs, Alison Shilton, Amanda Dowling, Anna Holland, Anna Kemsley, C. Green, Cara Gambon, Catherine Bressington, Charlie Thompson, Christina Longman, Cindy Port, Claire Smith-White, Danielle Perkins, Deidre Hussey, Di Wyeth, Fiona Allison, Florence Chauyara, Frances Warner, Gifty Dadzie, Hannah Brown, Helen Hawkins, Irene Tan, J. Willard, Jackie White, Jaimie Sutherland, Jannine Bailey, Jayne Moss, Jenny Evans, Jessica Main, Jo Green, Joanna Broomham, K. Wren , Karen Plews, Karen Spencer, Karen Wrigley, Kasia Russel, Katie Harrald, Kirsty Fisher, Laura Kirk, Lauren Barnett, Lauren Bartlett, Leah Pueschel, Lisa Rudall, Liz Treen, Louise Wylie, Melanie Taylor, Michelle Chuter, Michelle Hardy, Michelle Mcloughlin, Michelle Nicholls, Naomi Davies, Nicola Wimshurst, Pat Harris, Paula Ball, Penny Schnabel, R. Warner, Rachel Bright, Rachel Rouen, Rebecca Beddows, Ruth Beesley, Sabine Everett, Sarah Heath, Saras Bishop, Sham Shelbourn, Sharon Phipps, Shian Fethney, Sophie Adams, Sophie Hutton, Susan Meyjes, Tabitha Stuthridge, Tania Gaffney, Theresa Thomas, Tina Longman, Tracy Hopkins, Vicky Donovan, Vivienne Novis, Wendy Bascal, Zoe Farr;

Gloucestershire Royal Hospital: Louis Khor (Principal Investigator), Angela Smith, Angela Stevens, Bryony Bell, C. Pearson, Carly Avis, Caroline Broadhurst, Cassy King, Chantel Coleman, Charlotte Barwick, Cody Allen, Ellen Reeves, Fiona Liddle, Hayley Marvill, Jane Barradale-Smith, Jane Soule, Jenna Surman, Jennifer Pratley, Kerrie Lotsu, Lisa Frattolillo, Louise Broadley, Lucy Broad, Michelle Dimery, Michelle Partington, Nikki Dobson, Nina Kellow, Rachael Harris, Rachel Midwinter-Marland, Ridwana Pandor, Sarah Pilcher, Sian Harrington, Tracey Miller; *Great Western Hospital, Swindon:* Tracey Sargent (Principal Investigator), Amy Pitcher, Angela Bunce, Anita Long, Ashley Heal, Danielle Heywood, Debra Hunt, Denise Selby, Elaine Price, Jade Gordon, Jane McGregor, Jennie Hone, Joanna Coulson, Joanne Lewis, Julia Sewell, Kate Welsh, Kathryn Owen, Kelly Greenslade, Kimberley Tubb, Lisa Nicholson, Nicola George, Rachel Ravati, Rebekah Tollafield, Ruth Davies, Sara Brown, Sophie Stewart, Susan Bint, Tamara Byrne, Tanya Miles, Victoria Norman, Viv Cutler, Zhilla Majadabadi; *Hillingdon Hospital:* Jane Terry (Principal Investigator), Denise Ahmed, Emma Speirs, Fiona White, I. R. Howarth, Kirsty Griffith, Kirsty Stark, Licricia Ngahan-Tchaptchet, M. Lawlor, Manjit Matharu, Sarah Bell-Ryan; *James Paget University Hospital, Great Yarmouth:* Mumtaz Rashid (Principal Investigator), Andrea Bedford, Angela Oram, Ann Pye, Caroline Fox, Elsie Gibbs, Emily Boyce, Emily Cole, Faye Hewitt, Helen Cullimore, Jane Ward, Jennifer Thompson, Joan Timewell, Kerry Burwood, Laura Jarrett, Lauren Goodfellow, Lesley Yates, Nora Hassan, Pauline Studley, Sinead Osborne, Sophie Neville, Tracey Porter, Victoria Reeve; *King's College Hospital, London:* Cathy Walton (Principal Investigator), A. Tully, Agnes Kimbowa, Alice-Amanda Hinton, Amelia Evans, Anna Mazzarelli, Bridget Rance, Bryanna Chenoweth, C. Beckmann, Christine Murphy, Clare Patterson, Clemmie Hooper, Dorcas Appah, Dorisilla Adolwa, Emily Stockton, Erica White, Erika Glenny, Esther Annan, Halina Szajna, Iqra Khan, Katherine Clark, Katrice Currie, Kelly Macfadyen, Laura Santos, Mary Bollard, Mary Obud, Michelle Lynch, Modupe Adebayo, Olivia Snowball, Omatalani R. Sangare, Rachael Waldron, Rachel Barlow, Rebecca Macleod, Rebecca Manners, Ruth Graham, Ruth Landis, Sadie Holland, Sarah Skivens, Sarah-Ann Evans, Sophie Halton-Nathan, Sophie Steward, Stacey Robinson, Sue Byrne, Susie Urquhart; *Kingston Hospital:* Arezou Rezvani (Principal Investigator), Alexandra Frost, Alice Cox, Amanda Carey, Amisha Chauhan, Anne-Marie Greaves, Annie Stott, B. Hellmich, Bronwen Kenward, C. Caton, Charlotte Rose, Chloe Du Parcq, Elka Dimitrova, Fiona Smith, Hui Tam, Jana Durtova, Jennifer Slee, Joanna Pitcher, Laura Grainger, Laura Sowell, Leanne Bateman, Lizzanne Roman, Louise Jones, Lucy Bellinger, Lyndsey Smith, Morwenna Trevan, N. Haysum, Nicky Ni, Nicole James-Lowe, Nikki West, Perrine Dhaisne, Rachel Bell, Rachel Rolfe, Rebekah Hoadley, Rosemary Mukasa, Ruth Sentenga, Sam Frewin, Sarah Lowe, Scarlett Beland-Tyce, Sophie Wismer, Suesan Beirouty, Susan Leahy, Susan O'Callaghan, Toni Brown, V. Gunawardena, Zandra Rubia-Mendoza; *Lewisham Hospital, London:* Frances Jones (Principal Investigator), Ellen Madamombe, Jade Johnson, Kay Holford, Mabinty Leigh Sian Turner, Suzannah Sheerin; *Medway Maritime Hospital, Gillingham:* Dorothea Smith (Principal Investigator), Andrea Curling, Belinda Newman, Deborah Simmons, Debra Nwosa, Dorothy Smith, Helen Jones, Jane Simmons, K. Ashwell, Kerry Sturgess, Lovelace Oti, Ludmila Wilson, Lynn Deller, Michelle Hayes, N. Jones,

Patricia Chaplin, Sarah-Jane Cross, Sharon Small, Tatjana Molotkova, Valerie Andrew, Zoe Wood; *Nevill Hall Hospital, Abergavenny:* Louise Taylor (Principal Investigator), Amanda Lisle, Andrea Priddle, B. Markey, Cath Barwise, Deb Oliver, Kath Barwise, Kerry Owen, Kim McKie, Leanne Ball, Mandy Jones, Pippa Nicholas, Sue Jordan, Wendy Howells-Smith; *New Cross Hospital, Wolverhampton:* Gowri Simon (Principal Investigator), Becci Leathley, Claire Morgans, Deborah Brettle, Ellen Poniatowska, Emma Clews, Hazel Peden, Hazel Remmet-Booth, Jane Hussellbee, Joanne Ridley, Julia Icke, Karen Evans, Laura Brooks, Louise Wood, Marni Fassett, Pip Grocott, Siva Basra, Tracy Willetts; *North Manchester General Hospital:* Viv Dickinson (Principal Investigator), Andrea Kerti, C. Sinclair, Catherine Holt, Catherine Hughes, Colette Robinson, Collette Riley, Dawn Littler, E. Foley, Ellie Cardnell, Emma Baxter, Emma Groom, Emma Park, G. Charles, Georgina Cartridge, Helena Spencer, Jean Davis, Jenna Haycocks, Jo Jakubowski, Joanna Ward, Julie Ainsworth, Julie Whitby, Karen Robbins, Kate O'Hagan, Kerri Delaney, Laura Ashton, Laurene Mannix, Lileath Fisher, Louise Blinkhorn, Lyndsay Yates, Lynsay Ingham, Margaret Kerins, Michelle Salt, Pam Whittle, Rachel Tully, Sacha Jackson, Stella Oakes, Tina Affleck, Valerie Julie Walker, Zoe Davies; *Pinderfields Hospital, Wakefield:* Nicolas Akerman (Principal Investigator), Anna Warburton, Caroline Paterson, Chiew Poskitt, Rachel Stock, Muhammad Faisal Ehsan, Elise McShee, Jacqueline Edge, James Bedford, Jill Greenwood, Julie Wormstone, Karen Simeson, Laura King, Marie Knox, Michelle Mowbray, Penny Barker, Rosalind Morley, Stacey Dunn, Sue Winrow, Tracy Langcake, Ujala Ahmad, Veronica Walker; *Prince Charles Hospital, Merthyr Tydfil:* Liz Edwards (Principal Investigator), Bev Jones, Catherine Bush, Ceri Hill-Jones, Diane Lewis, Jodie Hodges, Theresa Jones; *Princess of Wales Hospital, Bridgend:* Sarah Fox (Principal Investigator), Angela Davies, Annette Jones, Christie-Ann Lang, Donna Hall, Elinor Taylor, Elizabeth James, Emily Grainger, Gemma Griffiths, Hannah Lambert, Joy James, Julie Roberts, Kate Richards, Kathryn Greaves, Lauren Yaw, Lynne Grieves, Mari Davies, Megan Cave, Michelle Bassett, Rachel Morgan, Sarah Jones, Sian Middleton, Tracey Bowman; *Queen Alexandra Hospital, Portsmouth:* Aneeta Sinha (Principal Investigator), Amy Pollard, Andrea Gray, Ann Going, B. Edge, Beryl Pullen, Carol Richardson, Carole Longley, Ella Edwards, Ellie Jenkins, Emma Connelly, Emma Kellagher, Fiona Moore, Genevieve O'Docherty, Gill Allen, Isla Campbell, Isobel Murtagh, Jemma Cave, Jill Hall, Jo Jordan, Jo Warwick, Jodie Carolan, Karen Darr, Karen Munks, Karen Wellspring, Katherine O'Mara, Katrina Walker, Kim Leonard, Laura Davis, Linda Lishman, Lucy Galloway, Lulu Russell Smith, Lynda Baker, Lynne Groves, Mandy Whittle, Mary Taylor, Mary-Ann Sheehan, Melanie Say, Mo Turnbull, Naomi Simpson, Penny Bone, Penny Cox, Sally Griffiths, Sarah Burr, Sharon Buttriss, Suzanne LeBrocq, Tracey Hall, Tracey Lasisi, Vanessa Garlish, Wendy Bessant, Wendy Marsh, Zoe Garner; *Queen Charlotte's & Chelsea Hospital, London:* Felicity Platt (Principal Investigator), Alice Gautreau, Grace Kember, Igbeka Hayes, Karen McArtney-Roberts, Lisa Rickwood, Lucy Simpson, Suzanne Ridley; *Queen Elizabeth Hospital, Kings Lynn:* Anoop Surendran (Principal Investigator), Beverley Golding, Caroline Tucker, Catherine Bent, Debby Ramsdale, Donna Allen, E. Cervi, Emma Chapman, Helen Parker, Jacinta Baptista, Jean Keen, Jodie Cully, Jodie Jupe, Lisa Gormley, Liz Tyler, Michaela Bouskova, Naomi

Seaman, Rachel McCabe, Rosie Hucklesby, S. Tennant, S. Wingfield, Sarah Russell, Tracey Stafford, Tracy Cooke, Yvonne Fulcher; *Royal Cornwall Hospital, Truro:* Nila Cota (Principal Investigator), Amy Dunstan, Charlie Fulcher, Dariel Rowe, Eddi Theedham, Jane Parke, Jane Stubbs, Jenny Heron, Jo Bennett, Josie Dodgson, Julie Wallis, Karen Needham, Katherine Holland, Kerry Youngman, Kim Hewlett, Kimberly Fanson, Laura Quinn, Lisa Marchetti, Lizzie Cowan, Lorraine Kennedy-Snaith, M. Hobson, M. Underwood, N. P. M. Middleton, Samantha Broughton, Sarah Grigg, Suzanne Bryant, Tracey Rowe, Victoria Bassett, Wendy Preen; *Royal Gwent Hospital, Newport:* Louise Taylor (Principal Investigator), Amie Cook, Beth Comben, Carmen Rubio-Batanas, Chloe Rowsell, Donna Crocombe, Donna Fleming, Elleanor Griffiths, Fay Smith-Warren, Fiona Carter, Helen Bishop, Jane Morgan, Janet Lawson, Jessica Waters, Karen Halford, Lesley-Ann Bushell, Margot Jones, Michelle Haggart, Naomi Martin, Nicola Smith, R. Green, Rose Thomas, Sophie Savigar-Jones, Tara Welch, Tracey Griffiths; *Royal Hallamshire Hospital, Sheffield:* Vicky Wilson (Principal Investigator), Alison Morison, Alison Norris, Amanda Muller, Amy King, Anne Hemingway, Benash Nazmeen, Caroline Dabinett, Carollynn Jones, Carolyn Metcalfe, Cheryl Popovich, Claire Craine, Claire Sayan, Clara Mwatati, Clare Hennessey, Clare Lord, Clare Newton, Dalia Peretz, Deborah Cresswell, Faith Tye, Gill Hunt, Hannah Tebbs, Heather Croft, Helen Frow, Holly Hickman, Jessica Brookes, Jill Parton, Jo Varley, Joy Herdman, Judy Chang, Julia Thackray, Julie Hawksworth, Justine Todd, Kate Fish, Kathleen Farrand-Green, Kay Crowch, Laura Asher, Laura Chadwick, Leanne Rutkowski, Louise Roberts, Lucy Bon, Medeline Mudehwe, Melinda Pagden, P. Pokorna, R. Nye, Rebecca Bustani, Rebecca Weston, Rio Cooper, Rosie Barker, Sally Dawn, Samantha Young, Sara Calow, Sarah Bell, Sarah Senbeto, Sarah Swift, Steven Mackie, Sylwia Szarwark, Tracy Hobson, Victoria Lee, Victoria Wilkins, Wendy Few, Wendy Murphy, Zoe Riley; *Royal Sussex County Hospital, Brighton:* Vanessa Fludder (Principal Investigator), Alanna Dunkerton, Emma Peck, Helen Williams, Kate Clark, Rosheen Baker; *Royal United Hospital, Bath:* Tracey Sargent (Principal Investigator), Angela Fitzpatrick-Nash, Anne Moffatt, Anne White, Annie Collingwood, Ashley Heal, Bridget Dack, Camilla Hawkett, Charley Reschwamm, Christie Harrison, Cindy Stamp, Donna Williams, Ellie Grant, Elly Doyle, Emily Craig, Emma Tanner, Gemma Day, Hannah Cross, Hannah Jewell, Hannah Reid, Helen-Marie Crooks, Hilary Paice, Jane Norris, Jemma Freegard, Jennifer Reid, Jenny Pullen, Jo Waldron, Jo Woodburn, Julia Grant, Karen Doran, Kate Boulton, Katherine Jackson, Kathryn Vosper, Kathy L Holford, Katie Gooding, Kerry Perkins, Kim Miles, Laura Friend, Leah Harrold, Linda Davis, Liz Norton, Martina Gray, Mirella Popescu, Naomi Bonett-Healy, Nora Seager-Wilkendorf, Rachel Brierley, Rachel Coleman, Rebecca Lamb, Rebecca Murdoch, Rebecca Pendry, Rebecca Walsh, Rhian Motean, Rose Jenkins-Hunt, Ruth Branson, Sara Burnard, Sara Driver, Sarah Marks, Sasha Cairns, Sharon Seager, Susan Collins, Tamara Carr-Gomm, Tina Coffey, Tracy Boakes, Wendy Duberry, Wendy Giles-Smart; *Singleton Hospital, Swansea:* Sarah Fox (Principal Investigator), Amanda Bates, Cath Harris, Danielle Clifton, Ellie Brown, Felicity Curtis, Julie Ellerton, Julie Thomas, Kate Phillips, Kim Hillier, Linda Richards, Lisa Rees, Lucie Warren, Nicky Court, Outi Morris, Rachel Williams, Rebecca James, Rebecca Lewis, Sarah Fox, Sharon Cooling, Sharon Evan, Sian Phillips, Vicki Lennon; *South Tyneside*

Hospital, South Shields: Shamma Al-Inizi (Principal Investigator), A. MacKay, Allison Nicholson, C. Greaves, Delia Brennan, Emma Hindes, Helen M Parker, Judith Black, Linda McNamee, Louise Nicholson, Nicola Tindall, Shelley Bowie, Stacey McFarley; *St George's Hospital, London:* Asma Khalil (Principal Investigator), Amy Ridout, Angel Segura, Astell Aikines-Aryeetey, Bridget Okereke, Carmen Martin Martinez, Christiana Appeah, Claire Davies, Cristina Perez, Danielle Holbrook, Dede Efueye, Elaine Sheehan, Eleonor Cowlard, Emma Corrigan, Emma Freeman, Emma McCheyne, Erin Hutchings, H. Gardner, Iona Hughes, Iryna Santoskkaya-Marsh, Isabel Aylward, Isabelle Cornet, Jessica Welham, Joyce Adu-Amankwah, Judith Mugerwa, Julia Plana Soria, Leila Zahedi, Lorena Santana Cardenosa, N. Karali, Natalie James, Ngozi Asika, Ojevwe Owereh, Ophelie Granger, Paula Lavandeira, Paulette Joy Palmer, Raquel Vives Font, Rosie Sands, Roxanne Vidal, Sarah Esegbona-Adeigbe, Silvia Campo, Steve-Samuel King-Inneh, Sylvia Zoldak, Zinab Jalloh-Conteh; *St Mary's Hospital, London:* Felicity Platt (Principal Investigator), Birima Darego-Wokoma, Leticia Alvarez, Margarita Lopez-Liesa, Sarah Pilgrem, Suzanne Ridley, Teresa Ribera; *St Thomas' Hospital, London:* Annette Briley (Principal Investigator), A. Perlepe, A. Veitch, Abby Stewart, Abosede Kako-Are, Agatha Okafar, Ailsa Gill, Alhan Javan, Alice Du Preez, Alison Armstrong, Amanda Stephens, Amy Davies, Amy Smith, Ana Elices, Ana Llamas, Anita Meagher, Ann Marie McHugh, Anna Dios, Anna Gaudion, Anna Kenny, Anne Cobell, Annie Bell, Beth King, Birima Darego-Wokoma, Blanca Rodrigo-Ibanez, Bree Cant, Camella Main, Dede Efueye, E. Blasse, Edith Onyeneri, Elena Martinez-Zuddin, Eleonora Bruni, Elizabeth Connelly, Elsa Moro, Emilie Grantham, Emily Jelen, Emma Copley, Emma Gray, Emma Grey, Emma Paten, Emmeline Mudford, Erika Duncan, Faye Safari, Florence Awichi, Fran Lawrence, Funso Adegoke, Gail Roberts, Gemma Baillie, Gillian Donaldson, Gloria Brempong, Grace Obwona-Lanana, Hannah Delmar-Addy, Hannah Emerson, Hannah Levy, Hannah Rogers, Hannah Veazey, Harriett Ivey, Hayley Osborne, Henrietta Simire, Holly Hickman, Ida Bradley, Iro Perlepe, Isabelle Arrabel, Jacqueline Mhako, Jane Love, Janet Cooper, Jennifer Tubby, Jess Cavaya, Jess Floyd, Jessica Quaroni, Jessima Cavaya, Jo Hoffmann, Jo McCarthy, Jo Parker, Jordan Anderson, Juliet Falola, Juliette Falolu, Karine Tweddle, Katia Ciccarella, Kaz Herlihy, Kylie Gould, Laura Bridle, Lauren Chandler, Lianne Phipps, Lianne Prior, Lola Shomefun, Louise Higgs, Lydia Gerrie, Madelena Wilders, Maeve O'Connell, Maggie Lee, Mara Bruno, Marcia Trusty, Maria Pipi, Marina Daniele, Marisa Alvarez, Marta Fernandez Diez, Monika Franklin, Moronike Agboola, N. Carlin, Namgyal Gonkatsang, Olivet Macfarlane, Olivia Wheeler-Robinson, P. Blair, Pauline Jackson, Rachel Grazette, Rosalind Pouteaux, Sarah Driver, Sarah Evison, Sarah Fowlie, Sarah Kensington, Sarah Tanner, Selina Tettey, Sharon Mumford, Sonia Pereina, Sophie Robinson, Stacy Brown, Stefania Andrian, Stella Nanseera, Sue Turner, Sumaira Bashir, Vaishni Moorji, Vic Offredi, Vivienne Gosden, Yemisi Fadoungbo, Yvonne Mcgrath, Zahra Famili, Zainab Jalloh, Zeenath Uddin, Zekiye Degmenlibey; *Sunderland Royal Hospital:* Kim Hinshaw (Principal Investigator), Amanda Bargh, Carol Forrester, Christine Evans, Claire Liddel, Deb Holmes, Deborah Bonney, Denise Mace, Donna Rodgers, Donna Rogers, Eileen Walton, Hannah White, Janet Rooks, Julie Harris, Julie Taylorson, Karen Hutchinson, Karen L. Armstrong, Kate Reedman, Kathryn

Evans, Katrina Dowell, Leeann Adey, Linda Adamson, Lisa Wilson, Lyndsey Summerbell,, Natalie Graham, Nicola Easton, Pam Cheek, Sheila Ford, Sonia Thompson, Sophie Robson, Stephanie Hepple, Suzanne Stelling, Victoria Young; *Tameside Hospital, Ashton-under-Lyne:* Gillian Singleton (Principal Investigator), Ann Gibson, Donna Saleh, Felicia Taylor, Gabby Greenwood, Gemma Lumley, Gillian Singleton, Helen Clase, Jackie Tomlinson, Jan Moriarty, Janet Danzi, Karen Rothera, Kate Firth, Kerry Jackson, Lisa Fisher, Louise Nelson, Paula Frazer, Rachel Drain, S. Bungaree, Sharon Aldous, Sophie Hook, Teresa Quinn, Tracey Leicester; *University College Hospital, London:* Belinda Green (Principal Investigator), Abisola Bashua, Amy Tiltman, Ann Esquerdo, Anna White, C. Amaning, Christine Haron, Constance Mvududu, Donata Hoesch, Edna Farah Dahir, Eleri Bates, Ellie Sanderson, Emily Nygaard, Fenya Jonas, Hayley Gilroy, Heidi Buhlmann, Ivan Bettinsoli, J. Cole, J. Evans, Jennifer Lang, Jenny Keys, Jes Surtees, Lucia Fitzsimons, Margarita Akyla, Meghan Jackson, Nellie Sarmiento, Teresa Okemadu, Zahra Khan; *University Hospital of Wales, Cardiff:* Rachel Collis (Principal Investigator), A. Morgan, A. Aimee Jones, Aime Symes, Alex Andrews, Alice Fairman, Alice Snell, Alyson Gardiner, Ami Wolstenholme, Amy Garrett, Amy Vaithilingam, Amy Welsh, Angela Amey-jones, Angela Jones, Anika Brodd, Anna Jones, Annie Kitchen, C. Swallow, Cara Moore, Cara Moruzzi, Carla Blackshaw, Catherine Downing, Cheryl Cox, Debbie Grey, Debbie Hunt, Debbie Jones, Deborah Powell, Ed Cross, Elaine Patterson, Elin Phillips, Emily James, Emily Shaw, Emma Bull, Felicity Callan, G. McElroy, Gloria Lane, Hannah Hills, Hannah Thomas, Helen Lock, Hollie Power, Jane Reid, Jayne Frank, Jenna Parsons, Jenna Terry, Jenny Rickson, Jess Holmes, Joanne Bowen, Jodie Clark, Jude Casey, Julia Morgan, Juliet Grimes, Karen Jennings, Karina Downing, Kate Lynch, Kate Murphy, Kate Siddal, Katherine Fischer, Katherine Williams, Kathryn Smith, Katie O'Bradovic, Katie Stubbs, Kelly Bennett, Kerri Hamblin, Kirsty Jones, Laura Mundy, Laura Rose, Laura Terry, Lauren Quirke, Lieska Hoes, Lindsey Hilldrup, Louise Houghton, Luisa Canale, Lynette Rowlands, Miranda Millett, Misha Harry, Natalie Rees, Nerys Kirtley, Nia Cleal, Nicola Savory, Nicola Schilling, Patricia Chan, Polly Ferguson, Rachel Bain, Rachel Harry, Rebecca Boselli, Ros Howells, Ruth Leonard, Sally Alqaddo, Sally Meek, Samantha Crouch, Sara Davies, Sarah Heap, Sarah James, Sarah Lucas, Sarah Madley, Sarah Morris, Sarah Spencer, Sherrie Bird, Shirley Goodwin, Sian Jones, Sofia Odugleh, Tamsin Edwards, Tracey Lawrence, Trudy Thomas-Jones, Wendy Hoggan, Zoe Millichap; *Warrington Hospital:* Rita Arya (Principal Investigator), Alison Quine, Ann Pathmakumar, Ann-Marie Brooke, Ann-Marie Hatton, Cate Fitzpatrick, Cath Kidd, Danielle Stotton, Deborah Fletcher, Debra Clements, Donna Abbott, Eileen Fielding, Einir O'Neill, Elaine Armitage, Hannah Stevens, Hayley Axon , Heather Mee, Helen Ling, Helen Poulton, Jackie Richards, Jayne Wright, Katherine Conquest, Kerry Jones, Lesley Hampson, Linda Hennon, Mags Odell, Marie Wheatley, Mary Cubbon, Mary Hornby, Simone Peters, Susan Evans, Tamsin Hawkins, V. Hodson, Vicky Littlewood; *West Middlesex University Hospital:* Louise Page (Principal Investigator), A. Akodu, Adebola Aroboto, Adelaide Adubuffour, Alicia Thomas, Alyson Brown, Amanda Bray, Anna Piasecka, B. Collard, B. Snee. C. Gordon-Jack, Deborah Reid, Eleanor Fraser, F. Addow, Fiona Ghalustians, Grace Volo, Hannah Thomas, Ilaria Torre, J. Fernandez, Jennifer Ryan, Jessica Howard, Joy

Ataderie, Julia Harris, Juliet Joseph, Karen Beck, Karen Lundie, Kelly Mack, Kirsty Dolling, Lisa Takab, M. Farrell, Maddie Saunders, Marie Garvey, Marie O'Connell, Marie Oliver, Mercy Batchelor, Neveen Jivan, Nikki Jaques, P. Laurence, Pia Tomeldon, Po Ying Li, R. Shadna, Renske van Gaans, Revai Chingwa, Risi Akodu, S. Harrison, Sally Dauncey, Sally Seaman, Sarah Dixon, Silviya Giffin, Sophie Pike, Tsakani Tshavane, V. Henry, W. Fambe.
